# S-palmitoylation Is Required for the Control of Growth Cone Morphology of DRG Neurons by CNP-Induced cGMP Signaling

**DOI:** 10.3389/fnmol.2018.00345

**Published:** 2018-09-24

**Authors:** Alexandre Dumoulin, Alina Dagane, Gunnar Dittmar, Fritz G. Rathjen

**Affiliations:** Max Delbrück Center for Molecular Medicine, Berlin, Germany

**Keywords:** cGMP signaling, S-palmitoylation, natriuretic peptide receptor 2, C-type natriuretic peptide, cGMP-dependent kinase I, growth cone morphology, axon branching

## Abstract

Genetic investigations have demonstrated that a specific form of axonal branching - the bifurcation of afferents from dorsal root ganglia (DRG), cranial sensory ganglia (CSG) and mesencephalic trigeminal neurons (MTN) – is regulated by a cGMP-dependent signaling pathway. This cascade is composed of the ligand C-type natriuretic peptide (CNP), the receptor guanylyl cyclase Npr2, and the cGMP-dependent protein kinase Iα (cGKIα). In the absence of any one of these components, axons no longer bifurcate, instead they turn in either an ascending or a descending direction. To gain further mechanistic insights into the process of axon bifurcation we applied different cell culture approaches to decipher downstream activities of cGKI in somatosensory growth cones. We demonstrate that CNP induces an enlargement of DRG growth cones via cGKI which is considered as the priming step of axon bifurcation in the spinal cord. This growth cone remodeling was both blocked by pharmacological inhibitors of S-palmitoylation and potentiated by blocking de-palmitoylation. cGKI colocalizes with the palmitoylome and vesicular structures including the endoplasmic reticulum, early endosomes, lysosomes primarily in the central domain of the growth cone as well as with the Golgi apparatus at the level of the soma. Interestingly, an acyl-biotin-exchange chemistry-based screen indicated that 8pCPT-cGMP-induced signaling induces S-palmitoylation of a restricted pool of proteins in the DRG-derived cell line F11. Overall, our data indicate that CNP-induced cGMP signaling via cGKI affects growth cone morphology of somatosensory afferents. Moreover, it also suggests that S-palmitoylation might play a role in this process.

## Introduction

During embryonic development navigating axons often form branches to build a complex and well-ordered network. Branch formation allows an individual neuron to establish contacts with distinct targets and thereby provide a framework for parallel processing of signals. Impairment of the neuronal network formation often leads to neurological disorders like schizophrenia (Bullmore and Sporns, 2009; Engle, 2010). Thus, unraveling molecular mechanisms regulating different axonal branching modes is of critical importance. A number of factors including extracellular cues, cytoskeletal components and signaling factors have been described to regulate axon branch formation (Schmidt and Rathjen, 2010; Gibson and Ma, 2011; Kalil and Dent, 2014; Armijo-Weingart and Gallo, 2017). However, the mechanism that drives branching at specific *in vivo* locations remains poorly understood.

**FIGURE 1:**
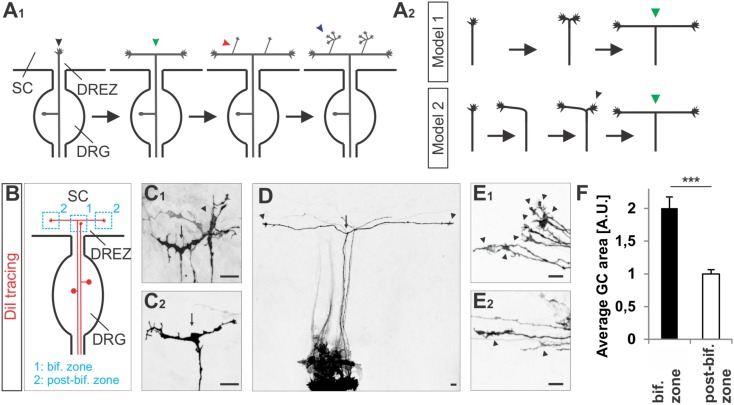
Growth cone splitting of somatosensory afferents at the dorsal root entry zone. **(A_1_)** Schematic illustrating the three major branching modes of somatosensory afferents in the spinal cord: bifurcation, collateral formation and terminal branching and **(A_2_)** the two possible options to build a T-shaped branch. **(B)** Schematic illustrating the sites at which individual sensory axons were analyzed at the DREZ of the mouse spinal cord. DRG afferents were labeled by the fluorescent lipophilic axon tracer DiI in the DRG and visualized at the DREZ. **(C_1_,C_2_)** Splitting growth cones in the bifurcation zone (growth cones within the DREZ that have not bifurcated yet) of the E12.5 mouse spinal cord. **(D)** A symmetric T-shaped DRG afferent with two growth cones (arrowheads) in the post-bifurcation zone from an E12.5 mouse embryo. The arrow depicts the bifurcation. **(E_1_)** Sensory growth cones (arrowheads) in the bifurcation zone of E12.5 mouse embryos. **(E_2_)** Sensory growth cones (arrowheads) in the post-bifurcation zone (growth cones that have already bifurcated and which are at least 100 μm away from the bifurcation point) of E12.5 mouse embryos. **(F)** Quantifications of growth cone areas in the bifurcation zone (*n* = 22, of these 3 were in the process of splitting whereas others were enlarged but not in a splitting conformation) and in the post-bifurcation zone (*n* = 34) from 3 littermate wild type E12.5 embryos. *p* < 0.0001 (unpaired two-tailed *t*-test), error bars represent SEM. Scale bar in all images, 20 μm. A.U., arbitrary unit; DREZ, dorsal root entry zone; DRG, dorsal root ganglion; GC, growth cone; SC, spinal cord.

The projection of dorsal root ganglion (DRG) afferents into the developing spinal cord is a very accessible and therefore useful system to investigate several types of axonal branching including bifurcation, interstitial and terminal branching (Brown, 1981). DRG central afferents first bifurcate at the dorsal root entry zone (DREZ) when entering the spinal cord. Then, after a waiting period they form collateral branches which sprout out from the axon shafts and invade the spinal cord in a dorso-ventral manner (Ozaki and Snider, 1997). Finally, they arborize in their termination fields in the superficial or deeper layers of the spinal cord where they form synapses (see also **Figure 1A_1_**) (Dumoulin et al., 2018). All three modes of axonal branching are thought to be activated by distinct signaling systems.

It was previously demonstrated that a cGMP signaling cascade regulates bifurcation – a specific form of axon branching – of DRG afferents at the DREZ of the developing mouse spinal cord (Schmidt et al., 2002, 2007, 2009; Zhao and Ma, 2009; Zhao et al., 2009; Ter-Avetisyan et al., 2014). This cGMP signaling cascade is composed of the ligand C-type natriuretic peptide (CNP) that activates the natriuretic peptide receptor 2 (Npr2, also known as NPR-B or GC-B), a guanylyl cyclase receptor which produces cGMP from GTP. Then, cGMP activates the cGMP-dependent kinase Iα (cGKIα, also called PKGIα) to phosphorylate so far unknown targets in sensory neurons. If one of these three components is missing in mouse mutants, sensory axons are not able to bifurcate and instead form either turns in rostral or caudal direction (Dumoulin et al., 2018). More recently, this signaling pathway was shown to be of equivalent importance for the bifurcation of afferents of cranial sensory ganglia (CSG) and of mesencephalic trigeminal neurons (MTN) within the developing hindbrain (Ter-Avetisyan et al., 2014; Ter-Avetisyan et al., 2018). Consequences of bifurcation deficits are altered termination fields of primary afferents from the skin in the spinal cord, impaired noxious heat perception, reduced nociception induced by chemical irritants and a reduced maximal biting force. Responses to mechanical stimulation and motor coordination are surprisingly normal in Npr2 mutants with bifurcation defects (Troster et al., 2018; Ter-Avetisyan et al., 2018).

Although cell culture experiments analyzing collateral branching or growth cone activities suggest that cGMP signaling regulates microtubule dynamics (Xia et al., 2013; Akiyama et al., 2016) the mechanism of axon bifurcation mediated by CNP/Npr2/cGMP/cGKIα is entirely unsolved. A multitude of actions including regulation of adhesion, modulation of the cytoskeleton (actin polymerization or organization of microtubules), trafficking of membrane proteins are possible and might be triggered downstream of cGKIα upon activation by CNP via Npr2. In the present study we aimed to better define the CNP-induced effects on DRG growth cones which are mediated by cGKIα. We used different cell culture approaches of wild type or cGKI-deficient DRGs to characterize the impact of the ligand CNP on growth cones. We show that CNP-mediated cGMP signaling induces an expansion of DRG growth cones and promotes neurite extension via cGKI which were both blocked by broad-spectrum inhibitors of palmitoylation. Pharmacological blocking of de-palmitoylation potentiated the effect of CNP, increasing the area of the growth cone. In DRG growth cones cGKI was found to be in close proximity to the palmitoylome and vesicular structures including the endoplasmic reticulum (ER). Accordingly, by an acyl-biotin-exchange chemistry approach we identified a small group of proteins in the DRG-derived F11 cell line that get palmitoylated upon 8-pCPT-cGMP stimulation. Overall, our findings demonstrate that S-palmitoylation might play a crucial role for sensory growth cone activities that are induced by cGMP signaling.

## Materials and Methods

### Mice

cGKI-deficient mice were genotyped as described elsewhere (Wegener et al., 2002). Animals were housed on a 12/12 h light/dark cycle with free access to food and water. The animal procedures were performed according to the guidelines from directive 2010/63/EU of the European Parliament on the protection of animals used for scientific purposes. All experiments were approved by the local authorities of Berlin (LaGeSO) (numbers T0313/97, G0370/13, and X9014/15).

### Cultures of F11 Cells and Embryonic DRG Neurons, Growth Cone Analysis

F11 cells were maintained in DMEM/F-12 medium supplemented with GlutaMAX^TM^ (Gibco, #31331028), 10% (v/v) FCS (Gibco, #16010167), 1% (v/v) HAT supplement (Invitrogen, #21060017) and 100 U/μL Penicillin(P)/Streptomycin(S) (Gibco, #15140122) at 37°C with 95% air and 5 % CO_2_. Overnight starvation was in the same medium without FCS.

Dorsal root ganglia single cells or explants were collected from E12.5 mouse embryos and from E7 chick embryos. For cell attachment assays DRGs from E13.5 mouse embryos were prepared to get larger number of cells. For monolayer or culture in a collagen matrix, DRGs were dissociated by trypsination for 20–30 min at 37°C in HBSS (Gibco, #24020091). Cells were dissociated with a fire polished Pasteur pipette and resuspended in 1 ml DMEM/P/S or DMEM/FCS/P/S (DMEM, Gibco, #61965). Dissociated cells were plated at 10,000 or 20,000 cells per well on laminin1 (10 μg/ml; Gibco, #23017-015) or 0.1 mg/ml poly-D-lysine (PDL) (Sigma, #A-003-M) coated 6-well Lumox^®^ dishes (Sarstedt, #94.6077.331) or 12-well glass slides (Ibidi, #81201) in DMEM supplemented with B27 (Gibco, #17504044), 10 ng/ml human β-NGF (Alomone labs, #N-245) and either 500 nM CNP or vehicle or in cell culture medium as indicated. Cells were incubated overnight at 37°C with 95% air and 5% CO_2_.

For neurite length measurements DRG explants or dissociated cells were cultivated in a collagen matrix. Two hundred microliter of a collagen mixture constituted of 1 mg/ml collagen (Collagen I, Rat Tail; Corning, #354236), 1x DMEM (from 10x DMEM, Sigma, #D2554), 0.5x F12 medium (from 1x F12 medium, Gibco, #31331028) and 0.8% NaHCO_3_ [from a 7.5% (w/v) stock solution in ddH_2_O, Sigma, #S5761] were added to a well (μ-Slide 8 well, IBIDI, #80826). Then, using forceps one DRG explant was placed into the center of each well. For single cell cultures, 40,000 cells were mixed with the aforementioned 200-μl collagen mixture and added to a well. Collagen gels were allowed to polymerize at 37°C with 95% air and 5% CO_2_ for 45–60 min followed by adding 200 μl of β-NGF/B27-containing culture medium as described above in the previous paragraph. Measurements of the neurite length of DRG monolayer cells or F11 cells was done with the help of the Simple Neurite Tracer plugin in ImageJ software (NIH Image, National Institutes of Health, Bethesda, MD, United States) (Longair et al., 2011). DRG cells attached to laminin1 or PDL were counted after fixation and staining with anti-NeuN, anti-L1CAM and DAPI from microscopic images using ZEN2011 software (Zeiss).

For the measurement of the growth cone area explants were cultivated overnight on PDL/laminin1 in DMEM/B27 supplemented with 10 ng/ml human NGF or as indicated and then incubated without or with 500 nM CNP (Sigma, #N8768) for 1 h. The incubation period of 1 h was deduced from imaging studies on branching *Zebrafish* axons (Ponomareva et al., 2014). Explants were gently fixed with PFA (Merck, #1040051000)/sucrose (Merck, #573113) in PBS (Wang and Marquardt, 2012) and stained for α-tubulin and phalloidin (F-actin) after permeabilization in 0.1% Triton X-100 (Merck, #1122981001) in PBS. Confocal images were taken with a 100x oil objective and the tracing tool in ZEN software blue edition (Zeiss) was used to trace the growth cone area. The beginning of the growth cone was defined by a broadening of the axon shaft from which on area measurements were started. As the general growth cone area varied from one independent experiment to another, growth cone areas were normalized by the average growth cone area of the control for each independent experiment. In experiments in which S-palmitoylation was modulated pharmacological reagents (2BP, Cerulenin, both from Sigma, #21604 and #C2389 or palmostatin B, Millipore, #178501) were simultaneously applied together with CNP.

The number of filopodia per growth cone was assessed using Zen software blue edition (Zeiss) and filopodia length using the Simple Neurite Tracer plugin in ImageJ software as described above for monolayer cells (Longair et al., 2011). Note that values were normalized to the average value of control for each independent experiment. Statistical analyses were performed with the Microsoft Excel 2010 software (Microsoft). P-values were calculated using paired and unpaired two-tailed Student’s *t*-tests.

Triple compartment neuron device (TCND500, Xona microfluidics) was used for compartmentalized culture experiments. Devices were attached to PDL pre-coated Lumox^®^ dishes (Sarstedt, #94.6077.331) and laminin1 coating was performed afterward as described by the supplier. A minimum of 90,000 E12.5 dissociated DRG neurons were given in the central channel (soma compartment) and neurons were incubated for 4–5 days at 37°C with 95% air and 5% CO_2_ in DMEM supplemented with B27 and 10 ng/ml human β-NGF (Alomone Labs, #N-245) until DRG axons consistently invaded both external channels (axonal compartments). Then, culture medium was removed from both axonal compartments and the same medium as above supplemented with 500 nM CNP or vehicle was given separately in each compartment and cells were incubated 1 h at 37°C with 95% air and 5% CO_2_ before fixation with PFA, 15 min at 37°C.

### Immunocytochemistry and Dil Axon Tracing

F11 or DRG cells were gently fixed 15 min at 37°C with pre-warmed 4% paraformaldehyde (PFA) in PBS and washed 3 times 5 min in PBS at room temperature. They were then incubated 4 min in PBS, 0.1% Triton X-100 at room temperature, washed 3 times (5 min each) with PBS and blocked 10 min in blocking buffer consisting of PBS, 1% heat-inactivated goat serum (Gibco, #16210064) or 1% BSA (Biomol, #01400.100). Cells were incubated with primary antibody in blocking buffer for 1 h at room temperature followed by washing and incubation with the secondary antibody at room temperature. For explant cultures staining with primary antibodies was performed overnight at 4°C and secondary antibodies 2 h at room temperature. Antibodies and their dilution are listed in **Supplementary Table S1**.

Analysis of colocalization was done by the Mander’s approach (Mander’s colocalization coefficient, MCC) (Dunn et al., 2011) to investigate the percentage of cGKI-positive pixels colocalizing to specific intracellular membrane marker (tM1) or vice versa (tM2). Threshold calculation using the Costes regression (Costes et al., 2004) was assessed by selecting a region of interest (ROI, growth cone or whole cell) and with the help of the coloc 2 plugin from the image processing package Fiji of the Image J software.

All images were captured with a Carl Zeiss LSM 710 NLO Laser Scanning Microscope equipped with ZEN 2010 software at a resolution of 1024 × 1024 pixels using the following objectives: a Plan-Neofluar 10x/0.30 NA objective, a Plan-Achromat 40x/1.40 NA oil objective or a Plan-Achromat 100x/1.40 oil DIC M27 objective (all from Carl Zeiss MicroImaging, GmbH). For the colocalization studies the pinhole was set at 115 μm. Confocal z-stacks (2.2 μm in depth) were recorded with a z-interval of 367 nm and assembled using Zeiss ZEN Blue edition software. All quantifications were performed in ImageJ. Images were imported into Corel Draw X3 (Corel) for uniform adjustment of contrast and brightness.

DiI axon tracing was performed as described previously for DRGs attached to the spinal cord dissected from E12.5 C57BL/6 embryos (Schmidt and Rathjen, 2011).

### Immunohistochemistry

Immunohistochemistry on PFA-fixed 15-μm-thick cryostat E12.5 spinal cord transverse sections was performed as previously described (Schmidt et al., 2002). Sections were blocked 1h at room temperature in a blocking buffer consisting of 1% inactivated goat serum, 0.1% Triton-X100 in PBS and stained overnight with the primary antibody at 4°C in the same buffer. Incubation with the secondary antibody was performed at room temperature in blocking buffer for 2 h. A list of antibodies used in this study and their dilutions is given in **Supplementary Table S1**. Note that whole embryos were fixed for 4 h at 4°C with 4% PFA in PBS and cryopreserved overnight in 30% sucrose in PBS at 4°C before embedding in O.C.T. compound (Tissue-Tek, Sakura, #SA62550).

### Bioorthogonal Labeling and Click Chemistry

Visualization of the palmitoylome in growth cones or F11 cells using bioorthogonal labeling and click chemistry was adapted from protocols as detailed elsewhere (Gao and Hannoush, 2014). Briefly, embryonic DRG or F11 cells were grown on PDL/laminin1 coated plates overnight and then further cultivated in culture medium supplemented with 100 μM 15-azidopentadecanoic acid (palmitic acid azide, Invitrogen, #C10265) or vehicle overnight. On the next day, cells were washed 3 times with pre-warmed PBS and then fixed 15 min at 37°C with pre-warmed 4% PFA in PBS. They were then washed 3 times 5 min in PBS at room temperature.

For click chemistry cells were permeabilized 4 min in PBS containing 0.1% Triton X-100 at room temperature and washed 6 times (5 min each) with PBS to remove the detergent. In each well, 100 μl of a solution containing 0.1 mM Biotin-PEG4-alkyne (Sigma, #764213), 1 mM TCEP [Tris(2-carboxyethyl)phosphine; Sigma, #C4706] and 1 mM CuSO_4_ (Sigma, #C1297) in PBS were added and cells were incubated in the dark at room temperature for 1 h (see scheme of the click reaction in **Supplementary Figure S5**). Cells were then washed 6 times with PBS. Palmitoylated proteins were visualized by streptavidin-Cy5. The optimal concentration of palmitic acid azide was established in pre-experiments using F11 cells (see **Supplementary Figures S5B_1_–B_3_**).

### Acyl-Biotin Exchange Chemistry and Biotinylated Proteins Pull-Down

To identify palmitoylated proteins F11 cells were stimulated with 1 mM of 8-pCPT-cGMP (Biolog, #C 009-10) for 10 min followed by subcellular fractionation. F11 cells were prepared by homogenization in 0.34 M sucrose supplemented with protease blockers [aprotinin (20 U/μl, Carl Roth, #A162), leupeptin (5 mM, Sigma, #L2884), pepstatin A (5 mM, Sigma, #P5318), PMSF (1 mM, Sigma, #P7626)]. Nuclei were pelleted at 200 × *g* for 10 min and the resulting supernatant was centrifuged at 100,000 × *g* for 10 min to obtain a crude membrane pellet and the cytoplasmic fraction in the supernatant. The membrane fraction was solubilized in 1% CHAPS (Merck, #1116620010) in PBS supplemented with protease blockers. Un-solubilized material was removed by centrifugation at 100,000 × *g* for 10 min. Palmitoylated proteins were then labeled by the acyl-biotin exchange (ABE) chemistry protocol as described by Wan et al. (2007). Free thiols were blocked by NEM (Thermo Scientific, #23030). Then thioester bonds between cysteines and palmitate were specifically cleaved by hydroxylamine treatment and finally newly free thiols were labeled by HPDP-biotin (Thermo Scientific, #21341) (see schematic in **Figure 6D**). Palmitoylated proteins were purified by streptavidin agarose pulldown (Thermo Scientific, #434341).

### Mass Spectrometric Analysis

Proteins were eluted from the beads using SDS-loading buffer. The eluted proteins were concentrated into one band and excised from the gel. The gel pieces were digested using an automated digestion setup (Kanashova et al., 2015). The eluted peptides from the in-gel digestion were resuspended in ammonium bicarbonate buffer. The peptide mixtures were separated by reverse-phase chromatography using an Proxeon nLC2 (Thermo Scientific, Dreieich, Germany) on in-house-manufactured 20 cm fritless silica microcolumns with an inner diameter of 75 μm. Columns were packed with ReproSil-Pur C18-AQ 3 μm resin (Dr Maisch GmbH). Peptides were separated using an 8 - 60% acetonitrile gradient (60 min) at a nanoflow rate of 250 nl/min. Eluting peptides were directly ionized by electrospray ionization and analyzed on a Q-Exactive plus mass spectrometer (Thermo Scientific). The mass spectrometer was operated in a data-dependent acquisition mode with dynamic exclusion enabled (30 s). MS1 (mass range 300–1700 Th) was acquired at a resolution of 70,000 with the ten most abundant multiply charged (z ≥ 2), ions selected with a 2 Th isolation window for HCD (Higher-energy collisional dissociation) fragmentation with a normalized collision energy of 30. MS2 scans were acquired at a resolution of 17,500 and injection time of 60 ms. The MS2 ion count target was set to 2 × 10^3^ and the max injection time was 300 ms. Only precursors with a charge state of 2–7 were sampled for MS2. Data were analyzed by MaxQuant software version 1.5.1.2. The internal Andromeda search engine was used to search MS2 spectra against a decoy mouse UniProt database containing forward and reverse sequences. The search included variable modifications of methionine oxidation and N-terminal acetylation, deamidation (N and Q) and fixed modification of carbamidomethyl cysteine. The minimal peptide length was set to seven amino acids, and a maximum of two missed cleavages were allowed. The FDR was set to 0.01 for peptide and protein identifications. Unique and razor peptides with a minimum ratio count of 1 were considered for quantification. Retention times were recalibrated based on the built-in non-linear time-rescaling algorithm. Statistical analysis was performed using the R software package^1^. Significant changes between the samples were determined using a two-sample *t*-test after imputation of missing values.

The mass spectrometry proteomics data have been deposited to the ProteomeXchange Consortium via the PRIDE partner repository with the dataset identifier PXD010326 (Vizcaino et al., 2016).

### RT-PCR

Reverse transcription was performed on RNA purified from either E12.3 mouse DRGs or E17 whole mouse embryos with RNeasy kit (Qiagen) and reverse-transcribed the same day into cDNA using the SuperScript^TM^ II Reverse Transcriptase kit (Thermo Scientific, #18064014). Primers and PCRs for the 3-hit candidates were designed using Primer3Plus (Bioinformatics group), Edit Sequ (DNASTAR) and MapDraw (DNASTAR) softwares. The following primers were used for amplification of specific cDNAs: Arhgef2 forward (5′-CTTAAAGGCTGGCTTCGTTG-3′), Arhgef2 reverse (5′-AGTCCAAGGGTAAGGCTGGT-3′), Atl2 forward (5′-GCTTGATGAAGAGGCTTTGG-3′), Atl2 reverse (5′-GCCAGTCAAGGGTTCATTGT-3′), Atp2c1 forward (5′-TCAGATGTGGCAAAGCAAAG-3′), Atp2c1 reverse (5′-TAACCAGCCAACCAACATGA-3′), Dad1 forward (5′-GCAGTTCGGCTACTGTCTCC-3′), Dad1 reverse (5′-GTGCTGGCAAAGAGGAAGTC-3′), Zdhhc3 forward (5′-CAGTAGATGGCACTGCAGGA-3′), zdhhc3 reverse (5′-TGTCTTGAGGCCTTTTTGCT-3′), Zdhhc13 forward (5′-AGCTGGTTCTAGCCTGGACA-3′), Zdhhc13 reverse (5′-AGCCCACAGGGTGATCATAG-3′), Gfpt1 forward (5′-ATCCCTTGGTGCCAGTGTAG-3′), Gfpt1 reverse (5′-TGCTGACCTGCATTTCTGAC-3′), Map4 forward (5′-ACCGTTTCAAAAGCCACATC-3′), Map4 reverse (5′-CCAGTGGGAGTGGTGTTTCT-3′), Mcoln1 forward (5′-AAACACCCCAGTGTCTCCAG-3′), Mcoln1 reverse (5′-ACCAGCCATTGACAAACTCC-3′), Ndst1 forward (5′-TGCCAGTGTGTTTTCTCTCG-3′), Ndst1 reverse (5′-ATGGCTGTTAGTGGGACAGG-3′), Rps16 forward (5′-TGAAGCCTCCAAGAAGGAGA-3′), Rps16 reverse (5′-ACAAAGGTAAACCCCGATCC-3′), Slc25a13 forward (5′-TGCTCTTAGCCGGTGCTATT-3′), Slc25a13 reverse (5′-GAGCAGCAACTCCTTTCCAC-3′), Tars forward (5′-CCATGAACTTAGCCCTGGAA-3′), Tars reverse (5′-GCAAACTGCTCCTTCTCCAC-3′).

## Results

### Sensory Axon Bifurcation at the DREZ of the Spinal Cord Is Carried out by Growth Cone Splitting

To get further insights into the CNP/Npr2/cGMP/cGKI-mediated branching we analyzed somatosensory afferents when entering the spinal cord (**Figure 1A_1_**). Two options might be conceivable to form a T-shaped branch at the DREZ: (1) splitting of the growth cone or (2) by the budding of a branch from an axon that had already turned either into an ascending or a descending longitudinal track (scheme in **Figure 1A_2_**). Visualization of individual sensory axons at the bifurcation zone by labeling of DRG neurons with the lipophilic axon tracer DiI revealed growth cones that either seem to equably segregate in two halves or appear as a symmetric structure like a T (**Figures 1C_1_,C_2_**). In addition, two branches of somatosensory afferents of equal length growing in opposite direction in the lateral margin of the cord were frequently detected (**Figure 1D**). Therefore, a self-evident interpretation might be that the two stem axons result from the equable splitting of the growth cone. These observations might exclude a process by which the incoming afferent first turns into the longitudinal track and another branch hereafter emerges as a collateral branch at the turning point to extend in the opposite direction of the longitudinal track. Moreover, in the bifurcation zone sensory growth cones are twice as large as during longitudinal extension at the lateral margin of the spinal cord (**Figures 1E_1_,E_2_,F** and see scheme in **Figure 1B**).

These changes in the morphology of the growth cone might involve addition of membrane material and/or reorganization of cytoskeletal elements. Therefore, to better define the process of bifurcation we studied DRG growth cones in the presence of the ligand CNP from wild type and cGKI-deficient mice and characterized the localization of cGKI in growth cones.

### CNP/Npr2-Mediated cGMP Signaling via cGKI Induces an Expansion of the Growth Cone Area of Cultured Embryonic DRG Explants

**FIGURE 2:**
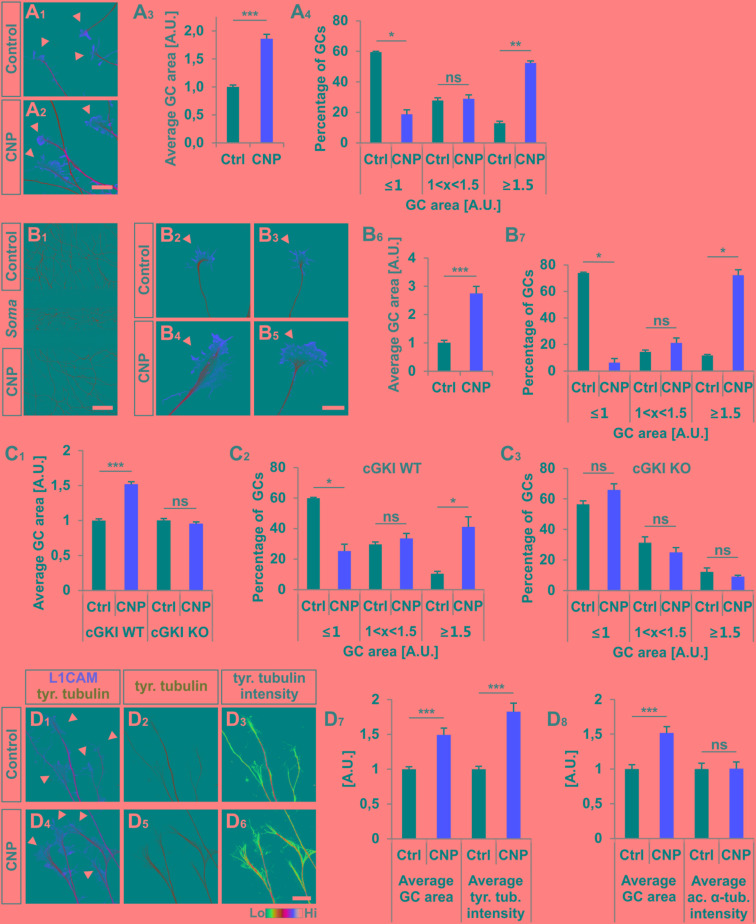
CNP induced a growth cone enlargement via cGKI. **(A_1_–A_4_)** E12.5 mouse DRG explants were grown for 16–20 h on laminin1 followed by treatment with (500 nM) or without CNP for 1 h. For visualization cultures were stained for α-tubulin (green) and F-actin (red). Arrow heads in A_1_ and A_2_ indicate growth cones. Scale bar, 20 μm. **(A_3_)** Quantification of growth cone area in confocal images using ZEN Blue edition software. Control *n* = 258 growth cones; CNP *n* = 180 growth cones from three independent experiments. **(A_4_)** Percentages of growth cone areas that were classified into three groups according to their area. Growth cones were compared to the normalized area value of the control. They were either equal or smaller than the average value 1 (≤1, small growth cone), up to 50% larger than this value (1 < x < 1.5, medium growth cones) or equal or larger than 50% of this value (≥1.5, large growth cones). **(B_1_–B_7_)** Similar results on growth cone enlargement of E12.5 mouse DRG dissociated cells were obtained using a three microfluidic chamber culture system in which CNP was applied to one axonal compartment and the second axonal compartment served as control. Cell somata are in the middle chamber. **(B_6_,B_7_)** Quantification as described for **(A_3_,A_4_)** (green neurofilament-M). Control chamber *n* = 87 growth cones; CNP-containing chamber *n* = 65 growth cones, three independent experiments. Scale bar in **(B_1_)** 500 μm; in **(B_2_–B_5_)** 20 μm. **(C_1_–C_3_)** CNP-induced enlargement of the area of growth cones requires cGKI (E12.5 DRG explants). **(C_2_,C_3_)** Growth cones from E12.5 wild type (WT) and cGKI-deficient mice treated with CNP or left untreated were classified into three groups as described in A_4_. cGKI wild type control *n* = 480 growth cones; cGKI wild type with CNP *n* = 319 growth cones; cGKI knockout control *n* = 250 growth cones; cGKI knockout with CNP *n* = 349 growth cones. Four independent experiments. **(D_1_–D_8_)** CNP-induced growth cone enlargement is accompanied with an increase of tyrosinated tubulin. **(D_1_–D_6_)** Control and CNP treated growth cones from E12.5 mouse DRG explants were stained with anti-tyrosinated tubulin and counterstained with anti-L1CAM. Scale bar, 20 μm. **(D_7_,D_8_)** Quantification of tyrosinated and acetylated α-tubulin. Tyrosinated tubulin: *n* = 118 growth cones (control) and 49 growth cones (CNP), three independent experiments; acetylated tubulin: *n* = 69 growth cones (control) and 73 growth cones (CNP), three independent experiments. *p* < 0.0001 (^∗∗∗^), *p* < 0.001 (^∗∗^), *p* < 0.05 (^∗^), or *p* > 0.05 (ns), unpaired **(A_3_,B_6_,C_1_,D_7_,D_8_)** and paired **(A_4_,B_7_,C_2_,C_3_)** two-tailed *t*-test. Error bars represent SEM. A.U., arbitrary unit; GC, growth cone; KO, cGKI knockout; WT, wild type.

To unravel the effect of the ligand CNP on Npr2-positive DRG growth cones and to better understand the function of cGKI we cultivated embryonic DRG neurons on extracellular matrix proteins in the presence or absence of CNP for 1 h. [This incubation period was deduced from imaging experiments of *Zebrafish* axons that bifurcate in the periphery (Ponomareva et al., 2014)]. We observed that CNP increased the growth cone area of cultured E12.5 mouse DRG explants compared to control (**Figures 2A_1_–A_3_**, arrowheads) after an incubation period of 1 h. Quantification of the average growth cone area showed that CNP increased the area up to 86% ± 8% (mean ± SEM) compared to control (**Figure 2A_3_**, *p* < 0.0001). As the growth cone population was heterogeneous, we classified growth cones in three populations according to their area normalized to the control average area: small (area ≤ 1 [A.U.]), medium (area between 1 and 1.5) and large (area ≥ 1.5) (Krylova et al., 2002). This evaluation revealed that CNP stimulation reduces the number of small growth cones (19% ± 3%, mean ± SEM) compared to control (59% ± 1%, mean ± SEM, *p* < 0.05) whereas it strongly increases the number of large growth cones (52% ± 1%, mean ± SEM) compared to control (13% ± 1%, mean ± SEM, *p* < 0.001) (**Figure 2A_4_**). Note, that similar results were found using E7 chick DRG explants demonstrating that this effect is conserved among both species (**Supplementary Figures S1A_1_–B_2_**). The increase in growth cone area was accompanied with a decline in the length of filopodia on growth cones, however, their numbers per growth cone remain unchanged in cultured E7 chick DRG explants (**Supplementary Figures S1C_1_,C_2_**).

Interestingly, the increase of the growth cone area was also induced in a three-compartment cell culture system when CNP was only added to one axon containing chamber while the second axon compartment served as control (**Figures 2B_1_–B_7_**). This indicated that the CNP-mediated signal is transduced at the level of the axon presumably at the growth cone.

To demonstrate that growth cone enlargement induced by CNP is mediated by cGKI – the downstream effector of CNP/Npr2 in DRGs – we analyzed DRG explants from cGKI-deficient mice in the presence or absence of CNP. In DRG explants from cGKI knockout the average growth cone area of CNP-treated DRGs was similar to the untreated culture (**Figure 2C_1_**, *p* > 0.05) and no significant difference was quantified for the distribution of small, medium or large growth cones (**Figures 2C_2_,C_3_**, *p* > 0.05). The majority of growth cones was small in both conditions (56% ± 2% in control and 66% ± 4% in CNP-treated samples, mean ± SEM) whereas a minority was large (12% ± 3% in control and 9% ± 1% in CNP-treated growth cones, mean ± SEM) (**Figures 2C_2_,C_3_**). This indicates that the CNP-triggered growth cone enlargement requires the cGKI.

In the applied *in vitro* culture systems we did not detect an increase of bifurcation of DRG axons in the presence of CNP as observed *in vivo* suggesting that additional signals present in the DREZ might be required to execute bifurcation. However, assuming that growth cone bifurcation is a complex, multi-step process, CNP-induced growth cone enlargement might be considered as the initial priming but compulsory step for bifurcation. Importantly, the CNP induced enlargement in growth cone area is accompanied with a significant increase of tyrosinated tubulin representing the dynamic tubulin pool. In contrast acetylated tubulin representing stabilized tubulin is not affected by the presence of CNP for 1 h (**Figures 2D_1_–D_8_**). Note that all culture experiments with DRG neurons were done in the presence of NGF indicating that only trkA-positive axons were studied. However, Npr2 as well as cGKIα are expressed in all embryonic DRG neurons suggesting that our observations might also apply to trkA-negative DRG axons (Schmidt et al., 2007; Ter-Avetisyan et al., 2014; Troster et al., 2018).

### CNP Increased Neurite Length and Attachment of DRG Neurons on Extracellular Matrices

**FIGURE 3:**
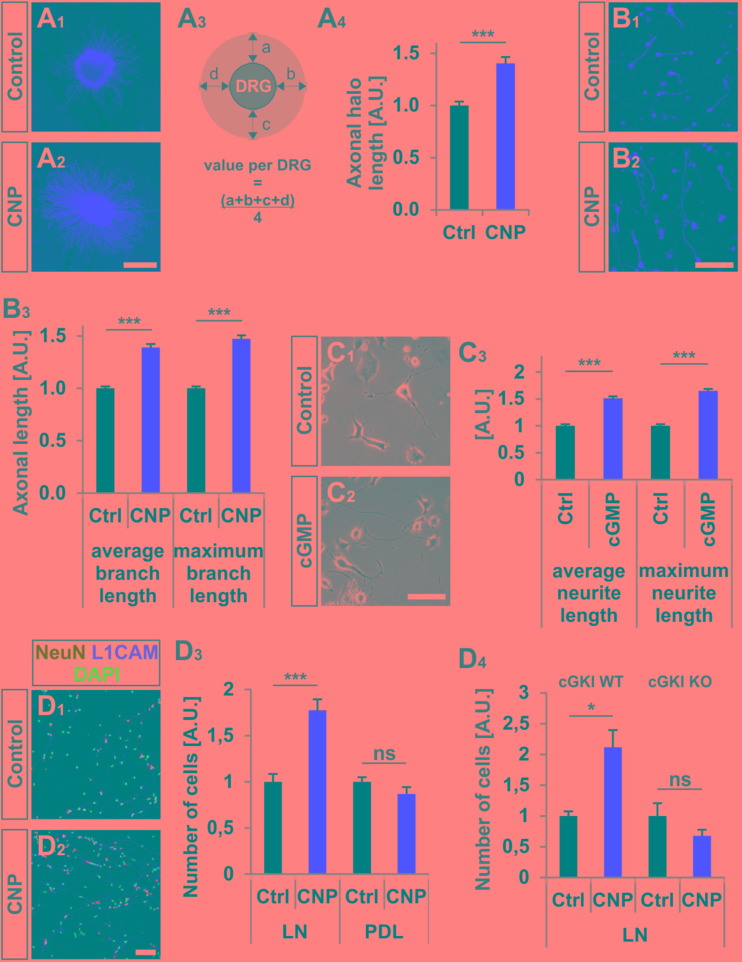
CNP increased neurite length and attachment of DRG neurons to extracellular matrices in culture. **(A_1_–A_4_)** Explants of E7 chick DRGs were grown in collagen matrix in the presence of 500 nM or absence of CNP for 16–20 h and stained with anti-NCAM1 for visualization. Scale bar, 500 μm. **(A_3_)** Scheme illustrating the calculation of the axonal halo from DRG explants (light gray). **(A_4_)** Quantification of the axonal halo from explants in the presence or absence of CNP. Control *n* = 12 explants; CNP *n* = 12 explants; three independent experiments; *p* < 0.0001 (^∗∗∗^), unpaired two-tailed *t*-test. **(B_1_–B_3_)** E7 chick DRG single cells cultured in a collagen matrix in the presence or absence of CNP for 16–20 h. Scale bar, 100 μm. **(B_3_)** Quantification of neurite length. Control *n* = 1671 cells; CNP *n* = 1077. **(C_1_–C_3_)** The length of neurites of F11 cells grown on PDL/laminin1 are increased by a cGMP analog (1 mM 8-pCPT-cGMP) treatment overnight. The average and the maximum lengths increased by 51 ± 4%, (mean ± SEM) and 65 ± 4% on PDL/laminin1, respectively. Three independent experiments *n* = 923 (control) and 1000 (8-pCPT-cGMP) cells measured *p* < 0.0001 (^∗∗∗^), unpaired two-tailed *t*-test. Scale bar, 100 μm. **(D_1_–D_4_)** Attached E13.5 mouse embryonic DRG cells on laminin1 or PDL after 16–20 h. **(D_1_,D_2_)** DRG cells were stained with anti NeuN, anti-L1CAM and DAPI and manually counted. **(D_3_)** Quantification of attached cells on laminin1 or PDL treated and untreated with CNP. **(D_4_)** Quantification of wild type or cGKI-deficient DRG neurons attaching to laminin1 in the presence or absence of CNP. Five independent experiments for wild type and 3 for knockout; *p* < 0.05 (^∗^). Scale bar in **(D_2_)**, 100 μm.

In addition to the growth cone enlargement, a long-lasting effect (16–20 h) of CNP was found on the neurite length as also described by (Zhao and Ma, 2009) and on the adhesion of DRG neurons to extracellular matrices. In a 3D collagen matrix CNP significantly increased the length of neurites from E7 chicken DRG explants or from dissociated cells, respectively (**Figures 3A_1_–B_3_**). In addition, an increased neurite length was also observed for DRG-derived F11 cells (Platika et al., 1985; Boland and Dingledine, 1990) on laminin1 in the presence of a cGMP analog (**Figures 3C_1_–C_3_**). Although F11 cells are derived from DRGs they do not express Npr2 at the protein level (data not shown) but do express cGKIα. Therefore a cGMP analog was applied to directly activate the kinase.

Attachment of dissociated E13.5 mouse DRG neurons identified by the neuronal marker NeuN and by L1CAM on the extracellular matrix protein laminin1 was increased of about 77% ± 12% (mean ± SEM, *p* < 0.0001) upon CNP stimulation after 16–20 h (**Figures 3D_1_–D_4_**). No rise was measured on the control substrate poly-D-lysine (**Figure 3D_3_**, *p* = 0.14) and attachment of DRG neurons was dependent on the presence of cGKI (**Figure 3D_4_**).

Overall, our data revealed that CNP induces a growth cone remodeling of cultured mouse and chicken embryonic DRG explants or dissociated cells leading to an enlargement within a period of 1 h with long-lasting effects on neurite length of chicken embryonic DRGs and attachment of embryonic mouse DRG neurons.

### cGKI Localizes in a Vesicular-Like Pattern Primarily in the Central Domain of Growth Cones of Cultured Embryonic DRGs

Since CNP-induced growth enlargement depends on the action of the cGKI we analyzed the localization of cGKI in growth cones of sensory afferents growing on laminin1 and in cryostat sections of mouse spinal cords by using a highly specific antibody to cGKI (Ter-Avetisyan et al., 2014). Cultured DRG neurons of cGKI-deficient mice are devoid of labeling demonstrating the specificity of the anti-cGKI antibody (**Supplementary Figures S2A_1_–D_3_**)

**FIGURE 4:**
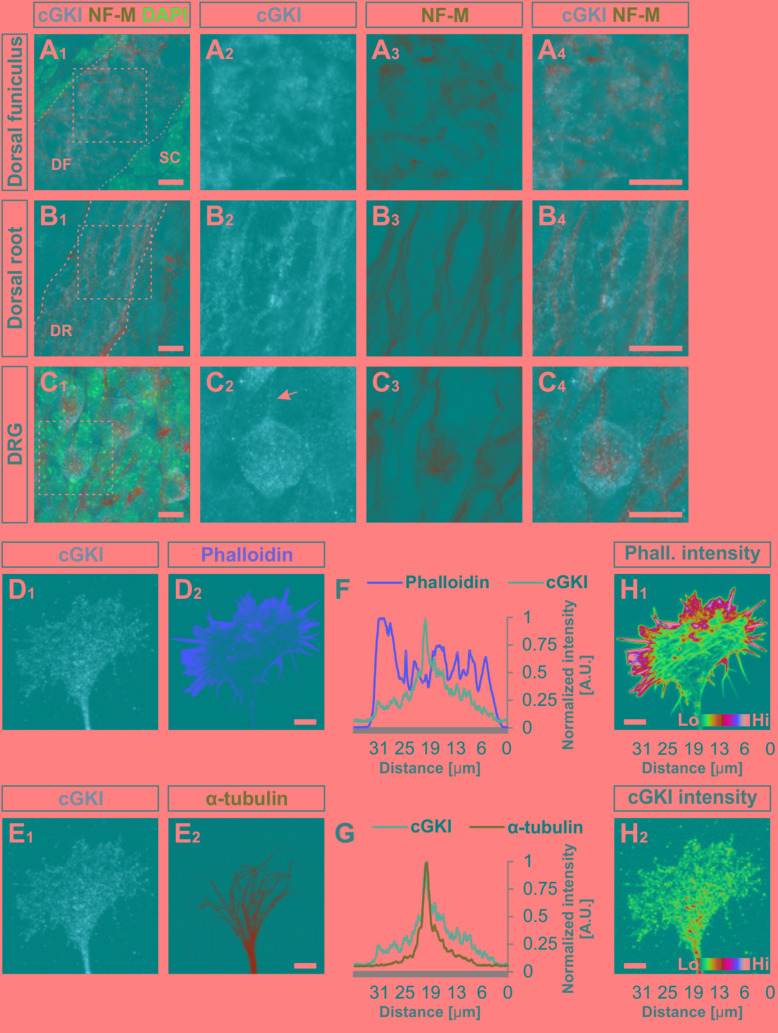
cGKI localizes in a vesicular-like pattern in sections of embryonic DRG neurons and in the central domain of cultivated DRG growth cones. **(A_1_–A_4_)** Transversal sections of the developing E12.5 mouse dorsal funiculus, **(B_1_–B_4_)** the dorsal root or **(C_1_ – C_4_)** DRG stained with anti-cGKI, anti-neurofilament-M or the nuclear stain DAPI. Higher magnifications of the squares indicated in **(A_1_,B_1_,C_4_)** are shown in **(A_4_,B_4_,C_4_)** respectively. Scale bar, 10 μm. **(D–G)** Localization of cGKI in growth cones of E12.5 DRG explants cultured on laminin1. **(D_1_,D_2_)** cGKI and F-actin (phalloidin). **(E_1_,E_2_)** cGKI and α-tubulin. Scale bar in **(D_2_,E_2_)** 5 μm. **(F,G)** Intensity profiles of a growth cone stained with anti-cGKI, F-actin (phalloidin) or α-tubulin summarized throughout the complete growth cone. Note that the intensity profile shown in **(F,G)** were taken from the entire growth cone. Along the *Y*-axis the intensity was summed up and shown along the *X*-axis (from the left to the right) using the profile tool in ZEN2011 software (Zeiss). **(H_1_,H_2_)** Pseudo-colored intensity pattern of phalloidin **(H_1_)** and cGKI **(H_2_)**. Scale bar 5 μm. Phall., phalloidin; Lo, low; Hi, high. A.U., arbitrary unit.

At embryonic stages cGKI is localized in a vesicular-like pattern throughout DRG cells as shown in transverse cryostat sections of the dorsal funiculus (**Figures 4A_1_–A_4_**), of the dorsal root (**Figures 4B_1_–B_4_**) and their somata (**Figures 4C_1_–C_4_**). In cultured DRG neurons cGKI appeared also in a vesicular-like pattern in the growth cone, the axon shaft and soma (**Figures 4D_1_,E_1_** and **Supplementary Figure S2A_2_**). For comparison, the staining phalloidin and of anti-α-tubulin of the same growth cone are shown which outline the F-actin-rich peripheral (P)-domain and the microtubule network of the central (C)-domain of the growth cone, respectively (**Figures 4D_2_,E_2_**). Intensity profiles of the localization of α-tubulin and of phalloidin over the complete growth cones indicated that cGKI is primarily localized within the C-domain of the growth cone contrasting the pattern of F-actin which is rich in the P-domain (**Figures 4F,G**). The pseudo-colored intensity pattern also clearly showed that vesicular cGKI is more concentrated in the C-domain of the growth cone (**Figure 4H_2_**) which is opposite to the F-actin (phalloidin) intensity pattern (**Figure 4H_1_**). Similar observations were made in DRG growth cones from the chick (**Supplementary Figures S3A_1_–B_2_**).

**FIGURE 5:**
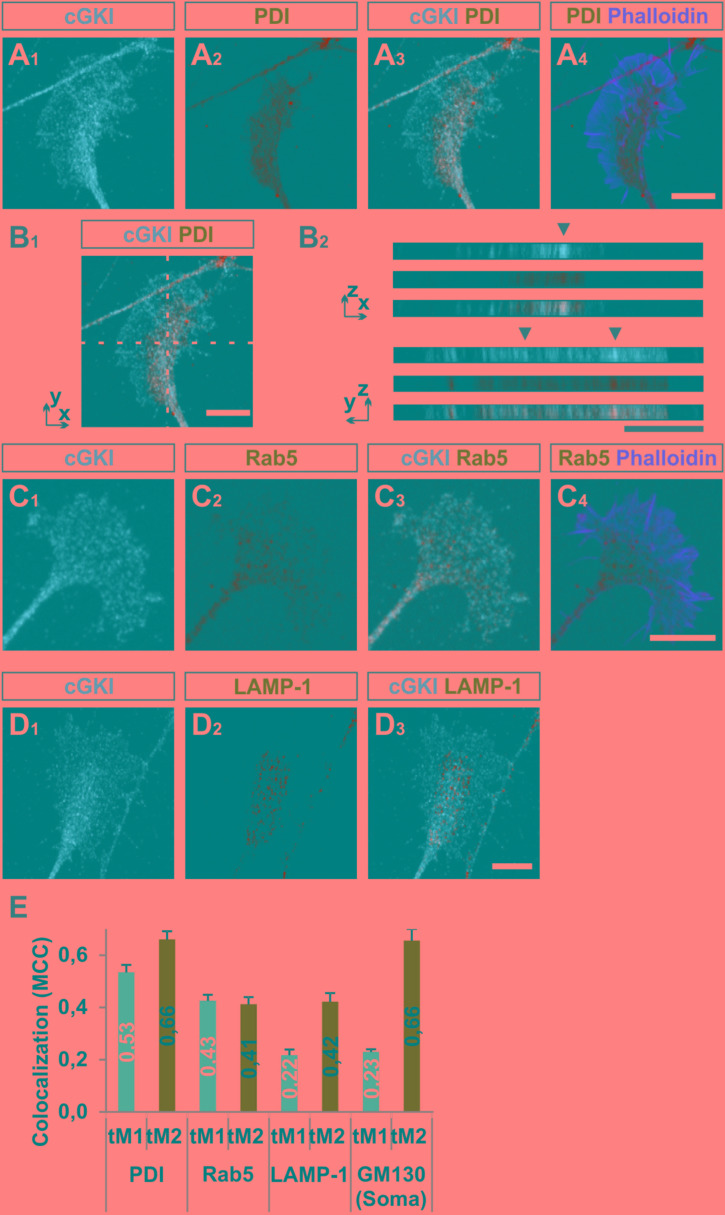
Colocalization of cGKI with marker proteins of intracellular compartments in DRG growth cones. **(A_1_–A_4_)** Colocalization of cGKI and the ER marker PDI in growth cones of E12.5 mouse DRG explants. A z-stack image was taken. For comparison the pattern of F-actin together with the localization of PDI is shown in **(A_4_)**. (Note: in the upper left corner an axon shaft is growing above the growth cone). **(B_1_,B_2_)** *z*-stacks taken at the dashed lines showing colocalizing cGKI and PDI (arrowheads). In **(B_2_)** *z*-stacks are presented as single colors or merged (lower panel). **(C)** Co-localization between cGKI and Rab5. F-actin is indicated by phalloidin staining. **(D)** Co-localization between cGKI and LAMP-1. **(E)** Quantification of co-localizations using Mander’s colocalization coefficient (MCC). MCC values are given within the columns. tM1 represents the proportion of cGKI-positive structures colocalizing with structures positive for a specific marker. tM2 represents the proportion of structures positive for a specific marker colocalizing with cGKI-positive structures. For each condition at least 10 growth cones or cell somata were analyzed PDI: 26, Rab5: 17, LAMP-1: 12 growth cones and for GM130: 10 somata. Error bars represent SEM. Scale bars, 10 μm.

To further characterize the subcellular localization of cGKI in sensory growth cones we performed double staining of anti-cGKI and different intracellular markers in cultured embryonic mouse DRG explants (except for GM130 for which dissociated neurons were analyzed). cGKI appears to colocalize partially with protein disulfide isomerase (PDI) which is primarily localized in the ER lumen (**Figures 5A_1_–A_4_**) and present within the C-domain of the growth cone (**Figure 5A_2_**). Visualization in the *z*-axis (dashed lines, **Figure 5B_1_**) clearly showed colocalization between cGKI and PDI (**Figure 5B_2_**, arrowheads). The colocalization within the growth cone was quantified using the Mander’s approach (Dunn et al., 2011). The Mander’ colocalization coefficient (MCC) calculation indicates a tM1 (cGKI versus PDI) of 0.53 ± 0.03 (mean ± SEM) and a tM2 (PDI versus cGKI) of 0.66 ± 0.03 indicating a colocalization of cGKI and ER structures in growth cones (**Figure 5E**). MCC calculations were also performed for Ras-related protein Rab-5 which is localized at the surface of early endosomes (**Figures 5C_1_–C_4_**), the lysosomal marker lysosomal-associated membrane protein 1 (LAMP-1, **Figures 5D_1_–D_3_**) which is a transmembrane protein localized at the surface of lysosomes and in the soma for the *cis*-Golgi marker 130 kDa *cis*-Golgi matrix protein which is present at its surface (GM130, not shown). MCCs calculation indicated a colocalization to different extents to cGKI, however, a tM2 value higher than 0.40 reveals a consistent presence of cGKI in structures positive for these markers (**Figure 5E**) (Dunn et al., 2011). In addition, when the entire cell was analyzed similar colocalization values were measured in the cultured cell line F11 which is derived from DRG neurons (**Supplementary Figure S4**). Here, cGKI was also found in a vesicular-like pattern throughout the cytosol and is strongly concentrated in the trans-Golgi compartment as well as in lysosomes, ER and in secretory vesicles positive for γ-adaptin.

Taken together, these results demonstrated that a substantial portion of cGKI is localized close to intracellular membranes including the ER, early endosomes and lysosomes primarily within the C-domain of the DRG growth cones and at the level of the soma in the Golgi apparatus compartment. The proximity to those structures suggests that cGKI might regulate the localization or activity of proteins found in the C-domain to induce growth cone enlargement and eventually the splitting of the growth cone *in vivo*.

### cGKI Colocalizes in the DRG Growth Cone With S-palmitoylated Intracellular Proteins

The vesicular-like localization of cGKI in the C-domain of somatosensory growth cones and its proximity to ER and Golgi structures prompted us to study whether the Npr2-mediated cGMP signaling cascade might regulate protein trafficking via S-palmitoylation in sensory growth cones. S-palmitoylation is a reversible post-translational modification that increases protein hydrophobicity and facilitates protein interactions with lipid bilayers. It is involved in the sorting and trafficking of intracellular proteins and might modify their function. The majority of palmitoyltransferases are localized in the ER and Golgi apparatus (Fukata and Fukata, 2010; Chamberlain and Shipston, 2015).

**FIGURE 6:**
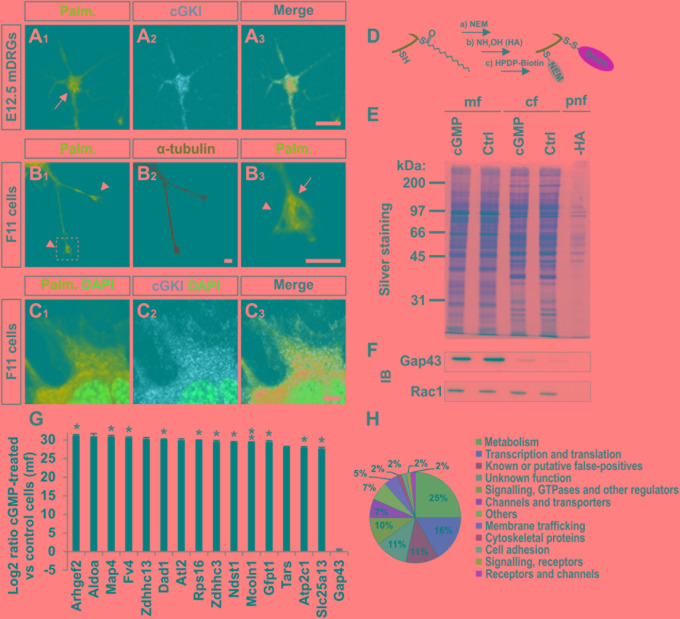
cGKI colocalizes with palmitoylated intracellular proteins in growth cones and 8-pCPT-cGMP-induced signaling stimulates S-palmitoylation of a restricted set of proteins. **(A_1_–A_3_)** Visualization of palmitoylated proteins by metabolic incorporation of a palmitate analog into proteins and by click chemistry in cultures of dissociated DRGs from E12.5 embryos followed by anti-cGKI staining. Palmitoylated proteins are present primarily in the C-domain (arrow) which overlaps with cGKI. Scale bar, 5 μm. **(B_1_–B_3_)** Visualization of palmitoylated proteins by the same approach in the center (arrow) of neurite-like protrusions (arrowheads) of F11 cells. For comparison the localization of α-tubulin is shown. The square in **(B_1_)** depicts the region enlarged in **(B_3_)**. Scale bar, 5 μm. **(C_1_–C_3_)** Visualization of palmitoylated proteins and localization of cGKI in the soma of F11 cells. Scale bar, 5 μm. **(D–H)** Purification of palmitoylated proteins upon 8-pCPT-cGMP stimulation by ABE chemistry. **(D)** Illustration of the labeling procedure of palmitoylated proteins by the ABE chemistry approach (Step a: thiol blockade with NEM, step b: thioesther bound cleavage with hydroxylamine (HA), step c: biotinylation of free thiols with HPDP-biotin moiety). **(E)** SDS-PAGE of palmitoylated proteins from F11 cells stimulated or non-stimulated (Ctrl) by 8-pCPT-cGMP (1 mM, 10 min). The post-nuclear fraction containing membrane and cytosolic proteins was used as control for the labeling procedure by omitting hydroxylamine (-HA). Molecular mass markers are indicated at the left of the panel. mf, crude membrane fraction; cf, cytosolic fraction; pnf, post-nuclear fraction. **(F)** Western blotting using anti-GAP43 and anti-Rac1 – two known palmitoylated proteins which were not enriched in the 8-pCPT-cGMP-stimulated fractions (Gap43 served as a positive control for the crude membrane fraction and rac1 for both cytosolic and membrane fractions). Note that no signal was detected in the sample without HA treatment revealing the specificity of the purification of S-palmitoylated proteins. **(G)** S-Palmitoylation of 15 proteins was increased upon cGMP stimulation in F11 cells in three independent experiments. Label-free LC-MS was used for quantification. Data are shown as log_2_ of the ratio between cGMP treated and untreated samples from the crude membrane fractions. 1 was given to control when it was equal to zero. Note that no change of S-palmitoylation for Gap43 upon cGMP stimulation was measured corroborating the Western blotting results in **(F)**. *p* < 0.05 (^∗^), *p* < 0.001 (^∗∗^). **(H)** Classification of proteins that were palmitoylated upon 8-pCPT-cGMP stimulation in 3 and 2 independent experiments (in total 60 proteins).

We asked to which extent palmitoylated proteins are present in the growth cone of cultured sensory neurons and whether these co-localize with cGKI. A cell culture approach consisting of the metabolic incorporation of a palmitate analog into palmitoylated proteins was applied which were then labeled by a click chemistry reaction with a biotin moiety (**Supplementary Figure S5A**) (Gao and Hannoush, 2014). This strategy allowed us to visualize the palmitoylome in DRG growth cones and cultured F11 cells by fluorescence microscopy (**Figures 6A_1_–C_3_**). Strikingly, palmitoylated proteins were omnipresent in the C-domain of growth cones of cultured mouse embryonic DRG neurons (**Figure 6A_1_**, arrow) and were also found to be enriched in tips of protrusions of F11 cells (**Figure 6B_3_**, arrow, 6C_1_ for the soma) as indicated by counterstaining for α-tubulin (**Figure 6B_2_**). These structures partially overlap with cGKI-positive structures within the C-domain of growth cones suggesting proximity between palmitoylated proteins and the kinase in growth cones and F11 cells (**Figures 6A_3_,C_3_**). MCC analysis demonstrated a tM1 of 0.84 ± 0.02 (SEM) and a tM2 of 0.73 ± 0.07 (SEM) between cGKI and the palmitoylome for F11 cells. Overall, our data might suggest a role of cGMP signal transduction on palmitoylation.

### 8-pCPT-cGMP-Induced Signaling Regulates S-palmitoylation of a Subset of Proteins in the DRG-Derived Cell Line F11

In order to investigate by biochemical approaches a possible link between cGMP signaling and protein S-palmitoylation we took advantage of the acyl-biotin-exchange (ABE) chemistry to purify S-palmitoylated proteins from cell lysates. In short, in this method free thiols are first blocked with *N*-ethylmaleimide (NEM), palmitates are then cleaved with hydroxylamine (HA) and new free thiols are labelled with a biotin moiety (HPDP-biotin, **Figure 6D**) (Wan et al., 2007). Since insufficient protein material was obtained from embryonic DRGs the F11 cell line was used.

We briefly stimulated (10 min) the cGMP signaling cascade in F11 cells with a cGMP analog (1 mM of 8-pCPT-cGMP), fractionated the samples and proceeded to the labeling of S-palmitoylated proteins using the ABE chemistry and a streptavidin-based pull down to purify biotinylated proteins. Hence, we purified the palmitoylomes from the crude membrane fraction (mf) as well as the cytosolic fraction (cf) of F11 cells stimulated or untreated with the cGMP analog as revealed by silver staining in a SDS-PAGE (**Figure 6E**). Note that the post-nuclear fraction (pnf) of unstimulated samples was used as a control in which the hydroxylamine (HA) treatment was omitted to identify proteins that were unspecifically biotinylated and/or pulled down (**Figures 6E**, -HA). The same samples were also immunoblotted for the known S-palmitoylated proteins such as the growth-associated protein 43 (Gap43) (Skene, 1989) or the Ras-related C3 botulinum toxin substrate 1 (Rac1) (Navarro-Lerida et al., 2012). No Gap43 or Rac1 signal was detected in the HA-negative control demonstrating the specificity of the ABE-based purification toward S-palmitoylated proteins (**Figure 6F**). GAP43 was found in the crude membrane fraction and Rac1 in the cytoplasmic fraction as well. Interestingly, label-free liquid chromatography–mass spectrometry (LC-MS) analysis of the membrane fractions revealed that stimulation by cGMP derivative 8-pCPT-cGMP resulted in an increased S-palmitoylation of 15 proteins (**Figure 6G**) which were consistently identified in 3 independent samples (3-hit protein candidates) stimulated by the cGMP analog but not in controls (unstimulated). Several of these proteins have also been detected in screens to be palmitoylated (see **Table 1** for the corresponding literature). Other palmitoylated proteins like Gap43 were purified from the crude membrane preparation in both samples (stimulated and unstimulated) at a similar level corroborating immunoblotting results shown in **Figure 6F** (log_2_ ratio close to 0). Moreover, a certain number of 2-hit candidates were found to be enriched in the 8-pCPT-cGMP-treated membrane fraction suggesting that this list of 15 candidates might be underestimated and together with 2-hit candidates forms a pool of about 60 proteins which get palmitoylated by 8-pCPT-cGMP-induced signaling and which are involved in various functions such as metabolism, transcription, signaling, membrane trafficking or cell adhesion (**Figure 6H** and **Supplementary Table S2**). mRNAs of 3-hit candidates were also shown to be expressed by RT-PCR in embryonic DRGs (**Supplementary Figure S6**).

**Table 1:** 3-hit palmitoylated protein candidates which were detected only in the F11 membrane samples stimulated with 8-pCPT-cGMP are listed.

Gene name	Protein name	Putative cGKI phosphorylation motif	CSS palm 3.0 (high threshold)	Literature (MS analyses)
Arhgef2	Rho guanine nucleotide exchange factor 2	RRRS;RKMT	Yes	Chesarino et al., 2014
Map4	Microtubule-associated protein 4	KKPT;KKVS;KRTS; KRPT;KRMT;KRNT	Yes	Yang et al., 2009; Wan et al., 2013; Chesarino et al., 2014; Thinon et al., 2016
Fv4	Retrovirus-related Env polyprotein from Fv-4 locus	KRAT	Yes	Chesarino et al., 2014
Zdhhc13	Palmitoyltransferase ZDHHC13	No	No	Li et al., 2012; Chesarino et al., 2014; Wei et al., 2014; Thinon et al., 2016; Shen et al., 2017; Sobocinska et al., 2018
Dad1	Dolichyl-diphosphooligosaccharide–protein glycosyltransferase subunit DAD1	No	No	Martin and Cravatt, 2009; Sobocinska et al., 2018
Atl2	Atlastin-2	RRQT	Yes	Shen et al., 2017
Rps16	40S ribosomal protein S16	RKKT	No	Martin and Cravatt, 2009; Forrester et al., 2011; Wilson et al., 2011; Gould et al., 2015; Serwa et al., 2015; Hernandez et al., 2016
Zdhhc3	Palmitoyltransferase ZDHHC3	No	Yes	Chesarino et al., 2014; Sobocinska et al., 2018
Ndst1	Bifunctional heparan sulfate *N*-deacetylase/*N*-sulfotransferase 1	KRLS	Yes	Li et al., 2012
Mcoln1	Mucolipin-1	RRAS;RRGS	Yes	Vergarajauregui and Puertollano, 2006; Li et al., 2012; Collins et al., 2017; Sobocinska et al., 2018
Gfpt1	Glutamine–fructose-6-phosphate aminotransferase [isomerizing] 1	KKGS;RRGS	Yes	Yang et al., 2009; Peng and Hang, 2015; Serwa et al., 2015; Thinon et al., 2016
Tars	Threonine–tRNA ligase, cytoplasmic	KKKS;KKET	Yes	Shen et al., 2017
Atp2c1	Calcium-transporting ATPase type 2C member 1	KRAS	Yes	Kang et al., 2008; Serwa et al., 2015; Collins et al., 2017
Slc25a13	Calcium-binding mitochondrial carrier protein Aralar2	No	No	Li et al., 2012; Gould et al., 2015; Hernandez et al., 2016; Shen et al., 2017


*Putative cGKI phosphorylation motifs were screened as well as putative S-palmitoylated cysteines using the CSS palm 3.0 software set on high threshold (Ren et al., 2008). Candidates were also screened whether they had already been purified in previous palmitoylated proteins screens using the “SwissPalm: Protein Palmitoylation database” (Blanc et al., 2015).*

Taken together, although side effects of 8-pCPT-cGMP cannot be excluded our biochemical results suggests that 8-pCPT-cGMP-induced signaling regulates the S-palmitoylation of a restricted pool of proteins in the DRG-like F11 cell line.

### S-palmitoylation Is Essential for CNP-Induced Growth Cone Enlargement and Neurite Extension

**FIGURE 7:**
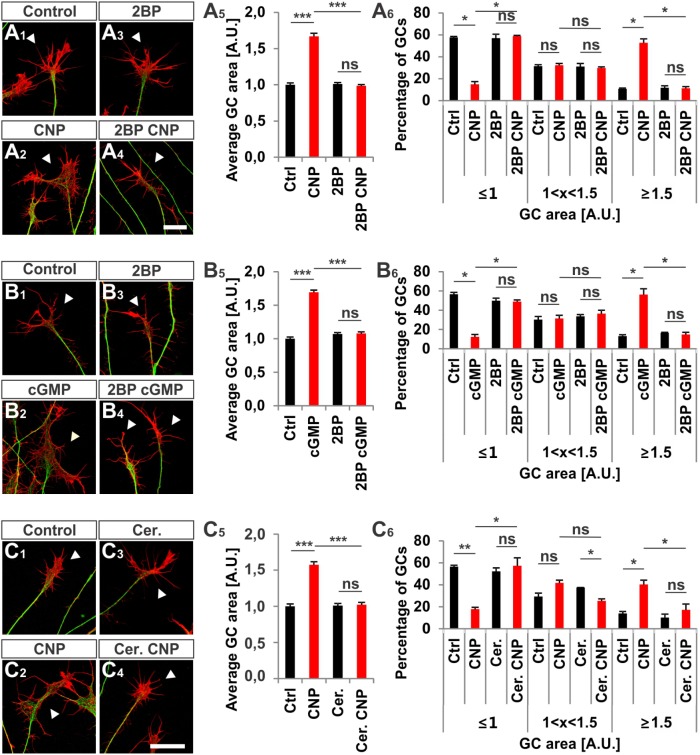
S-palmitoylation is required for CNP-mediated growth cone enlargement. **(A_1_–A_4_)** Control versus CNP-treated E7 chick DRG growth cones (explants) (arrowheads) in presence or absence of 20 μM 2BP and stained for α-tubulin (green) and F-actin (red). **(A_5_)** Quantification of the average area of growth cones treated as in **(A_1_–A_4_)**. Number of growth cones measured: 271 (control), 252 (CNP), 494 (2BP) and 482 (2BP CNP) from 3 independent experiments. **(A_6_)** Percentages of growth cones that were classified into three groups according to their area as introduced **Figure 2A_4_**. **(B_1_–B_4_)** Control versus 8-pCPT-cGMP (50 μM) treated E7 chick DRG growth cones (explants) (arrowheads) in presence or absence of 20 μM 2BP and stained for α-tubulin (green) and F-actin (red). **(B_5_)** Quantification of the average area of growth cones treated as in **(B_1_–B_4_)**. Number of growth cones measured in three independent experiments: 332 (control), 374 (cGMP), 382 (2BP) and 350 (2BP plus 8-pCPT-cGMP). **(B_6_)** Percentages of growth cones that were classified into three groups according to their area. **(C1–C6)** Control versus CNP-treated E7 chick DRG growth cones (explants) (arrowheads) in presence or absence of 20 μM Cerulin and stained for α-tubulin (green) and F-actin (red). **(C_5_)** Quantification of the average area of growth cones treated as in **(B_1_–B_4_)**. Number of growth cones measured in three independent experiments: 202 (control), 193 (CNP), 215 (Cerulenin), 266 (Cerulenin plus CNP). **(C_6_)** Percentages of growth cones that were classified into three groups according to their area. *p* < 0.0001 (^∗∗∗^), *p* < 0.001 (^∗∗^), *p* < 0.05 (^∗^), or *p* > 0.05 (ns), unpaired **(A_5_,B_5_,C_5_)** and paired **(A_6_,B_6_,C_6_)** two-tailed *t*-test. Error bars represent SEM. A.U., arbitrary unit; Cer., Cerulenin, Ctrl, control. Scale bars: 20 μm.

Next, we investigated whether S-palmitoylation and CNP-induced growth cone enlargement are linked in embryonic DRG neurons. Therefore, we took advantage of the broad spectrum S-palmitoylation inhibitor 2-bromopalmitate (2-BP) that blocks palmitoyltransferases (Chamberlain and Shipston, 2015) and applied it to cultured E7 chick DRG explants on PDL/laminin1. Remarkably, a low-dose of 2BP (20 μM) abolished the CNP-mediated expansion of the embryonic chick DRG growth cone without affecting the growth cone morphology when given alone (**Figures 7A_1_–A_4_**, arrowheads). Whereas CNP stimulation induced an increase in the average growth cone area of 67% ± 4% compared to control (mean ± SEM, *p* < 0.0001), the presence of 2BP inhibited the enlargement effect of CNP to control values (Ctrl and 2BP, *p* > 0.05) (**Figure 7A_5_**). A classification of growth cones into 3 populations according to area as described above showed that the CNP-based increase in large growth cones (53% ± 4% compared to 11% ± 1% in control, mean ± SEM, *p* < 0.05) (**Figure 7A_6_**) was reversed to control values by blocking S-palmitoylation (Ctrl and 2BP, *p* > 0.05) (**Figure 7A_6_**). The opposite was observed for the small growth cone population (≤1 A.U.). The presence of CNP decreased the percentage of small growth cones from 58% ± 1% (mean ± SEM) down to 15% ± 3% (mean ± SEM, *p* = 0.006) (**Figure 7A_6_**) whereas 2BP completely blocked the CNP effect by reaching 59% ± 6% (mean ± SEM, *p* > 0.05) (**Figure 7A_6_**). Note that the medium group of growth cones remained constant across all experimental conditions (around 30%, *p* > 0.05) (**Figure 7A_6_**). Importantly, 2BP application alone had no effect on the average growth cone area or on the growth cone area distribution compared to control (*p* > 0.05) (**Figure 7A_6_**). Similar results were obtained by applying 8-pCPT-cGMP (50 μM) instead of CNP indicating that S-palmitoylation is required downstream of cGKI and not upstream of Npr2 (**Figures 7B_1_–B_6_**).

Cerulenin that inhibits fatty acid and steroid biosynthesis (Baenke et al., 2013) and thereby interferes with S-palmitoylation also blocked the effect CNP on growth cone morphology of cultured E7 chick DRG explants to a similar extent as 2BP (**Figures 7C_1_–C_6_**). The average growth cone area in cultures treated with CNP and Cerulenin reached control values and also the classification of growth cones into three groups correlated with the control allocation. These results demonstrated that S-palmitoylation promotes CNP-mediated growth cone remodeling of cultured embryonic DRG explants.

**FIGURE 8:**
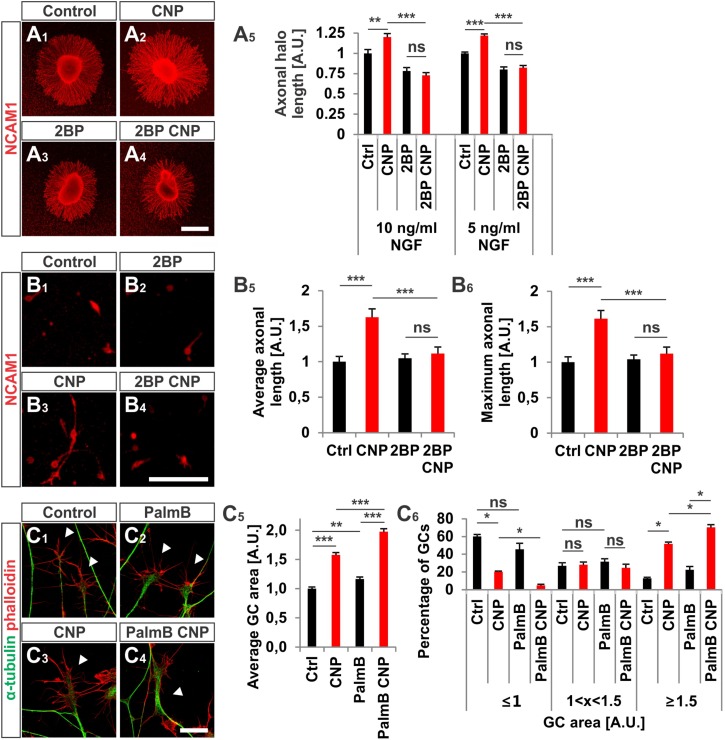
CNP-induced neurite extension is abolished by 2BP and growth cone enlargement is increased by interfering with acyl protein thioesterase 1 (APT1)-mediated de-palmitoylation. **(A_1_–A_4_)** E7 chick DRG explants were cultured overnight in a collagen matrix in either the presence of 500 nM CNP, 100 μM 2BP or 500 nM CNP plus 100 μM 2BP. Explants were then fixed and stained for NCAM1. **(A_5_)** CNP stimulation increased the axonal outgrowth of about 20% ± 4% (10 ng/ml NGF, mean ± SEM) and 22% ± 2% (5 ng/ml NGF, mean ± SEM). Blocking S-palmitoylation by 2BP inhibited the CNP-mediated axonal outgrowth increase. 100 μM 2BP itself inhibited axonal outgrowth compared to control (–22% ± 4% in 10 ng/ml NGF and –20% ± 3% in 5 ng/ml NGF, mean ± SEM). For measurement of axon outgrowth see also **Figure 3A_3_**. Number of explants assessed at 10 ng/ml NGF: 19 (Ctrl), 19 (CNP), 18 (2BP) and 18 (2BP plus CNP). Number of explants assessed in 5 ng/ml NGF: 13 (Ctrl), 15 (CNP), 16 (2BP) and 16 (2BP plus CNP). Three independent experiments per NGF concentration. Scale bar, 500 μm. **(B_1_–B_4_)** E7 chick DRG single cells were cultured overnight in a collagen matrix either in the presence of 500 nM CNP, 20 μM 2BP or 500 nM CNP plus 20 μM 2BP. Cells were then fixed and stained for NCAM1. Scale bar, 100 μm. **(B_5_,B_6_)** Quantification of the average and maximal axonal length showed that 2BP abolished the CNP-induced axonal length. Control *n* = 87 cells; CNP *n* = 114; 2BP *n* = 132; 2BP plus CNP *n* = 122. **(C_1_–C_4_)** Control versus CNP-treated (500 nM) E7 chick DRG growth cones (explants) (arrowheads) in the presence or absence of 20 μM palmostatin B and stained for α-tubulin (green) and F-actin (red). Scale bar, 20 μm. **(C_5_)** Quantification of the average area of growth cones treated as in **(C_1_–C_4_)**. Number of growth cones measured: 224 (control), 222 (CNP), 230 (PalmB) and 206 (PalmB CNP) in 3 independent experiments. **(C_6_)** Percentages of growth cones that were classified into three groups according to their area. PalmB, palmostatin B. *p* < 0.0001 (^∗∗∗^), *p* < 0.001 (^∗∗^), *p* > 0.05 (ns), unpaired **(A_5_,B_5_,B_6_,C_5_)** or paired **(C_6_)** two-tailed *t*-test. Error bars represent SEM.

2BP also reversed the long-lasting effect of CNP on the neurite length of E7 chick DRG explants or monolayers (**Figures 8A_1_–B_6_**). Blocking S-palmitoylation by using 2BP at a concentration of 100 μM significantly impaired the axonal outgrowth of embryonic chick DRG explants in a collagen gel matrix in the presence of either 10 or 5 ng/ml NGF cultured overnight (**Figure 8A_5_**). The relative axonal halo length of explants was significantly reduced to around 20% in both cases (*p* < 0.05 and *p* < 0.0001) (**Figure 8A_5_**). Similar results were obtained by applying CNP and 20 μM 2BP to dissociated E7 chick DRG monolayer cells in a collagen gel without any difference in the axonal outgrowth compared to control (**Figures 8B_1_–B_6_**).

As S-palmitoylation blockade prevents the CNP-Npr2-cGKI-mediated growth cone remodeling we tested whether the opposite - de-palmitoylation – affects DRG growth cone morphology. Thus, we challenged growth cones from E7 chick DRG explants with CNP together with 20 μM of the acyl protein thioesterase 1 blocker palmostatin B (PalmB) (Dekker et al., 2010). Blocking de-palmitoylation potentiated the effect of CNP on the growth cone enlargement compared to CNP alone (**Figures 8C_1_–C_6_**, arrowheads). The average area of growth cones treated with both CNP and PalmB was increased of about 97% ± 5% (mean ± SEM) compared to control whereas CNP alone increased the area of about 58% ± 4% (mean ± SEM, *p* < 0.0001) (**Figure 8C_5_**). Note that PalmB alone significantly increased the average growth cone area of about 16% ± 4% (**Figure 8C_5_**, *p* < 0.001). Consistently, PalmB and CNP together induced a decrease in the percentage of small growth cones down to 5% ± 1% (mean ± SEM) compared to the 20% ± 2% of CNP alone (mean ± SEM, *p* < 0.05) and an increase in the percentage of large growth cone up to 70% ± 3% (mean ± SEM) compared to CNP alone that was of about 52% ± 2% (mean ± SEM, *p* < 0.05) (**Figure 8C_6_**). Note that PalmB itself did not induce a significant change in the percentage of the growth cone population (**Figure 8C_6_**, *p* > 0.05). These results indicate that blocking de-palmitoylation potentiates the CNP-mediated growth cone remodeling and are in line with the experiments in which S-palmitoylation was blocked.

Taken together, our results suggest that S-palmitoylation might play a role downstream of cGKI in the CNP-mediated growth cone remodeling and propose that S-palmitoylation might be also important for axon bifurcation *in vivo*.

## Discussion

**FIGURE 9:**
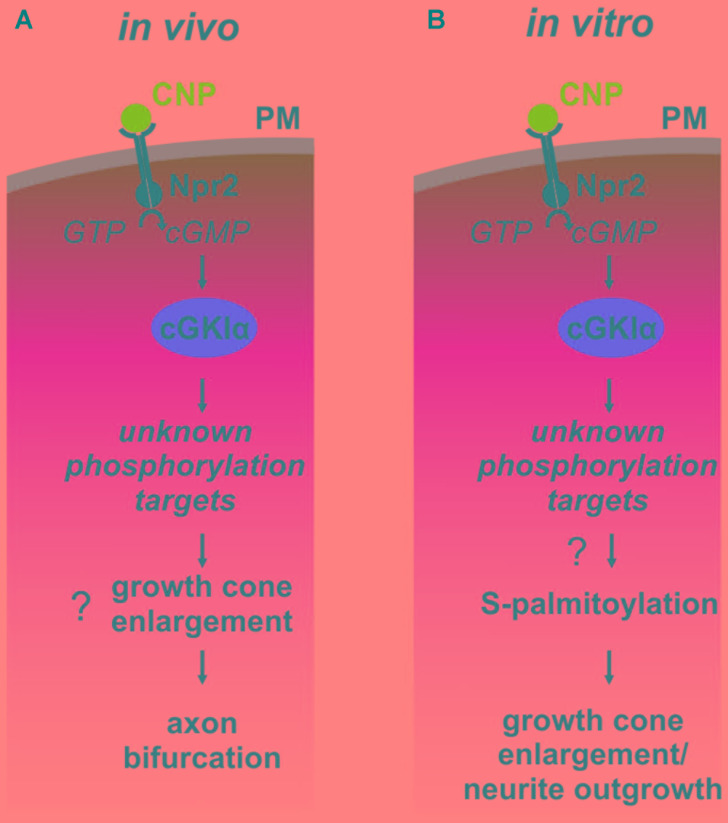
Illustration of the cGMP signaling cascade essential for bifurcation of somatosensory axons in the spinal cord/hindbrain and of growth cone shaping cell cultures. The growth cone enlargement induced by CNP in vitro is considered as priming step for bifurcation which requires S-palmitoylation. **(A)** *In vivo* CNP binds to its receptor Npr2 which generates cGMP from GTP. cGMP in turn activates cGKIα that phosphorylates so far unknown targets which are required for proper bifurcation and which might include growth cone enlargement. **(B)** *In vitro* the same signaling cascade is activated by CNP that mediates growth cone enlargement and increased in neurite outgrowth of DRG neurons. Our results indicated that downstream of cGKI S-palmitoylation might play a role in the remodeling of growth cones and in the increase of neurite outgrowth.

Previous publications have shown that a cGMP signaling cascade including the ligand CNP, the guanylyl receptor Npr2 and the serine/threonine kinase cGKI is implicated in the bifurcation of all somatosensory afferents in the developing spinal cord or hindbrain (for a review see Dumoulin et al., 2018). CNP expressed in the dorsal spinal cord (Schmidt et al., 2009) and in specific rhombomeres of the hindbrain at early embryonic stages (Ter-Avetisyan et al., 2018) binds to Npr2 on afferents on somatosensory neurons which in turn generates cGMP from GTP. Then, cGMP binds to cGKI and thereby induces a conformational shift that activates the enzyme to phosphorylate serine or threonine residues of protein targets which are currently unknown for the process of bifurcation (**Figure 9A**). In the absence of one of these signaling components in mouse mutants the bifurcation is completely disturbed. *In vivo*, genetic inhibition of cGMP signaling does not generally impair the ability of sensory axons to grow or to generate collaterals but specifically impairs their ability to bifurcate (Dumoulin et al., 2018).

To get further insights into the process of bifurcation of somatosensory axons in this study we initially visualized individual growth cones at the DREZ. A number of microscopic images from sensory afferents at the DREZ strongly argue for a splitting process of the growth cone to establish T-shaped branches. Interstitial branching by an afferent that had already turned into the longitudinal track of the spinal cord can be considered as an implausible mechanism to generate cGMP-induced T-branches (see also schematic of **Figure 1A_2_** which illustrates both options). Therefore, our data also suggest that beyond CNP, Npr2 and cGKI additional components implicated in the process of bifurcation still need to be identified in growth cones.

Signaling pathways are often compartmentalized by anchoring relevant components in larger complexes to ensure spatiotemporal control of signaling. Compartmentation is also essential to pick the correct target for phosphorylation. For protein kinase A – the closest homolog of cGKI - a number of AKAPs (for A kinase anchoring proteins) have been characterized which immobilize PKA into scaffolds of signaling complexes containing activators and substrates which are phosphorylated by PKA (Torres-Quesada et al., 2017; Ercu and Klussmann, 2018). cGKI is expressed as two isoforms, termed α and β, that differ in their first ∼100 N-terminal residues. These segments harbor a leucine zipper-like domain that directs homodimerization and interactions with anchoring proteins (Francis et al., 2010; Hofmann and Wegener, 2013). From these two forms the α-form is expressed in DRG neurons (Schmidt et al., 2002) which is translated from an alternatively produced cGKI mRNA. Evidence for scaffolds involved in spatiotemporal cGKI signaling is scarce – only a few have been defined for cGKIβ (Francis et al., 2010; Hofmann and Wegener, 2013) and even less for cGKIα (Yuasa et al., 2000; Wang et al., 2004). Our studies showed that a substantial portion of cGKI is associated with subcellular compartments in the C-domain of the growth cone and suggests that anchoring mechanisms for cGKI might exist.

Axon bifurcation is a complex cell biological action involving the coordination of several processes. In general it is thought that intracellular signals provoking changes of the growth cone morphology require the action of components of the cytoskeleton and/or a tight control of protein trafficking. These processes might function independently or might affect each other (Tojima et al., 2011, 2014; Winkle et al., 2016). Both, the pattern of localization of the cGKI in DRG growth cones and blocking experiments by pharmacological reagents suggest that CNP-induced signaling via Npr2 and cGKI might control the regulation of intracellular protein trafficking by modulating S-palmitoylation of proteins. A role for protein trafficking in the process of axon bifurcation was observed for the peripheral but not for the central branch of Rohon beard cells in *Zebrafish*. Knockdowns of calsyntenin – a kinesin adaptor implicated in intracellular trafficking – showed that it is required for both interstitial branching and growth cone bifurcation. It regulates in part the endosomal transport from the cell body to developing axons and branch points by organizing microtubule polarity. (Ponomareva et al., 2014; Lee et al., 2017). It is noteworthy that calsyntenin knockdowns also influence processes which control growth cone motility and growth cone volume of Rohon Beard cells. In human patients disruption of the gene encoding the zDHHC8 palmitoyltransferase contributes to schizophrenia and in a mouse knockout of zDHHC8 a reduced branching of axon collaterals was observed further indicating that S-palmitoylation and trafficking might play a role in the formation of neuronal circuits (Mukai et al., 2008, 2015).

Although bifurcation could not be induced in *in vitro* cultures of embryonic DRGs we demonstrated that CNP is critical for the shape of growth cones. Application of CNP induced a growth cone remodeling resulting in an enlarged area. This process might be considered as a priming step for growth cone bifurcation. It required the presence of cGKI indicating that the complete CNP/Npr2/cGKI signaling cascade is implicated in the broadening of the growth cone area. This CNP-mediated growth cone enlargement was blocked by broad-spectrum S-palmitoylation blockers and was potentiated in the presence of a thioesterase inhibitor suggesting that cGMP-stimulated S-palmitoylation of proteins might be implicated in growth cone shaping (**Figure 9B**). Long lasting effects of CNP or cGMP were observed on the neurite length as also described by Zhao and Ma (2009) and on the attachment of DRG and F11 cells to extracellular matrix components coated on culture dishes. These findings are in line with the adhesion of vascular smooth muscle cells induced by cGMP derivatives (Weinmeister et al., 2008). cGKI was found to be localized primarily in the C-domain of the growth cone in vesicular-like patterns and was associated with intracellular structures such as the ER, lysosomes, early endosomes and in the soma the Golgi apparatus where the majority of zDHHCs palmitoyltransferases are located (Fukata and Fukata, 2010; Chamberlain and Shipston, 2015). Importantly, cGKI is localized close to intracellular membranes that are enriched in palmitoyltransferases and might have a certain proximity to proteins undergoing S-palmitoylation. In comparison to the C-domain cGKI was only weakly expressed in the P-domain which is characterized by actin filaments. This faint localization of cGKI in the P-domain of the DRG growth cone suggests that the influence of the cGKI on the regulation of the F-actin cytoskeleton during the process of T-branching might be limited. This subcellular localization of cGKI in sensory growth cones is in line with observations on different cell types where cGKI was detected in the perinuclear region including colocalization with marker proteins of the ER and Golgi apparatus (Ives et al., 1980; Cornwell et al., 1991; Pryzwansky et al., 1995; Monks et al., 1998; Feil et al., 2002; Wang et al., 2004; Casteel et al., 2008; Kato et al., 2015). Furthermore, we observed a correlation between the location of cGKI and the palmitoylome in extending growth cones. Consistently, biochemical investigation using ABE chemistry demonstrated that cGMP signaling induced by 8-pPCT-cGMP might modulate S-palmitoylation of a restricted number of proteins in the DRG derived cell line F11. Further studies on palmitoyltransferases might substantiate this potential link between cGMP signaling and palmitoylation.

Interestingly, two palmitoyltransferases zDHHC3 and 13 were detected only in the palmitoylome fraction of 8-pCPT-cGMP-treated F11 cells. This might suggest two possibilities in a context of cGMP signaling: (1) either the enzymes get palmitoylated by another one which might lead to their activation/inhibition or they might traffic to other cellular compartments where they will be able to palmitoylate another pool of proteins or (2) this increase of palmitoylation might be taken as a possible increase in their auto-palmitoylation state. As the auto-palmitoylation state of palmitoyltransferases most likely correlates with their activation state (Fukata et al., 2004; Jennings and Linder, 2012; Chamberlain and Shipston, 2015), it might suggest that zDHHC3 and 13 get auto-palmitoylated under 8-pCPT-cGMP stimulation which might reflect an increase in their activity toward specific substrates. This also raises the question of how cGKI might regulate S-palmitoylation. It might phosphorylate target proteins which will get palmitoylated by specific palmitoyltransferases. It might also activate indirectly (via downstream targets) but also directly those enzymes. Interestingly, zDHHC5 has been reported to undergo endocytosis after phosphorylation by the Fyn kinase in hippocampal neurons (Brigidi et al., 2015) and zDHHC3 activity has been reported to be controlled by Src kinase in hippocampal neurons as well (Lievens et al., 2016). Therefore, it is conceivable that cGKI might phosphorylate specific palmitoyltransferases and modulate their activity or change their subcellular localization. Four enzymes contain a cGKI putative phosphorylation motif in their cytosolic domain (zDHHC1, 5, 8 and 23) and it will be interesting to know whether cGKI phosphorylates these palmitoyltransferases. Phosphorylation of specific proteins might also prevent palmitoylation. For example, a negative influence of phosphorylation on S-palmitoylation was found for phosphodiesterase 10A (Charych et al., 2010).

Currently, it remains a matter of discussion which of the palmitoylated components identified by using the F11 cells might be implicated in the regulation of sensory growth cone remodeling – if at all. Only a few options will be considered here. Palmitoylation might facilitate association of candidates with intracellular transport vesicles and might therefore guarantee efficient intracellular trafficking within the axon or growth cone. Candidates might also include proteins that regulate the organization of the microtubule cytoskeleton or of cell adhesion proteins. For example the Ras homolog family member A (RHOA) guanine nucleotide exchange factor ARHGEF2 is tethered by the dynein motor light chain DYNLT1 to the microtubule network (Meiri et al., 2012) and interacts with a number of proteins that are implicated in microtubule organization (Sandi et al., 2017). MAP4 has been shown to regulate the movement of pigment granules along microtubules to the cell center of Xenopus melanophores (Semenova et al., 2014). Therefore, MAP4 attached vesicles might promote the entry of new cargo into the expanding growth cone or MAP4 might be transported via dense core vesicles into the growth cone and after de-palmitoylation might stabilize the microtubule network in the growth cone. Such a mechanism has been demonstrated during axonal development for palmitoylated MAP6 which is present in secretory vesicles and the Golgi apparatus (Tortosa et al., 2017; Tortosa and Hoogenraad, 2018). A role of cGKI in tubulin dynamics which is linked to membrane trafficking to modulate growth cone area or shape has been also described by *in vitro* experiments (Xia et al., 2013). For the Ig superfamily adhesion proteins belonging to the SynCAM family (SynCAM4 – 2-hit protein candidate) an enlargement of sensory growth cones, very similar to that observed here, was demonstrated if they are offered as substrate in cell culture dishes (Frei et al., 2014). Other potential candidates for growth cone enlargement might be Mucolipin-1 (Mcoln1, also designated TRPML) or Ndst1 (*N*-deacetylase/*N*-sulfotransferase). The latter controls *N*-sulfation during heparin sulfate and heparin biosynthesis. Absence of Ndst1 affects several signaling pathways such as FGF, BMP, wnt and Hedgehog (Hh) which bind to heparin sulfate and play a role in growth cone extension (Pallerla et al., 2007; Ringvall and Kjellen, 2010). Interestingly, Mcoln1 - a lysosomal cation channel - is known to play a role in lysosomal exocytosis and vesicular transport to the plasma membrane (Venkatachalam et al., 2015). Moreover, the S-palmitoylation of its C-terminal tail leads to its association with membranes and most likely to its presence at the cell surface (Vergarajauregui and Puertollano, 2006) making this candidate attractive in the context of Npr2-mediated growth cone enlargement.

Overall, on the basis of the growth cone reshaping by CNP our results suggest that the CNP/Npr2/cGKI signaling pathway might control axon bifurcation in part through palmitoylation of selective proteins. In conclusion, our data on the localization of cGKI in a vesicular-like pattern in the central domain of the growth cone, on growth cone enlargement induced by CNP that paralleled with palmitoylation further our understanding of the process of bifurcation and establishes a potential link between cGKI-mediated phosphorylation and the regulation of palmitoylation.

## Author Contributions

ADu performed experiments and evaluated the data. GD and ADa performed the MS experiments and analyzed the MS data. ADu, GD, and FR contributed to the writing of the manuscript.

## Conflict of Interest Statement

The authors declare that the research was conducted in the absence of any commercial or financial relationships that could be construed as a potential conflict of interest.

## Footnotes

1 http://www.r-project.org

## Supplementary Material

FIGURE S1 CNP induced growth cone enlargement of cultured embryonic chick DRG explants. **(A_1_–A_4_)** 1-h CNP stimulation increased the growth cone area of E7 chick DRGs cultured on a PDL/laminin1 substrate. DRG neurons were fixed and stained for α-tubulin (green) and F-actin (red) (phalloidin). Control growth cones appeared with an extended P-domain containing long filopodia (arrowheads) whereas CNP-stimulated growth cones had a larger C-domain and shorter filopodia (arrowheads). **(B_1_)** The average area of CNP-stimulated growth cones increased 52% ± 6% (mean ± SEM) compared to control. **(B_2_)** The number of small growth cones was drastically reduced (19% ± 3%, mean ± SEM) compared to control (60% ± 2%, mean ± SEM) and the number of large growth cones was significantly increased (46% ± 2%, mean ± SEM) compared to control (12% ± 1%, mean ± SEM). **(C_1_)** The average length and maximum length of filopodia at growth cones were reduced upon CNP stimulation of about 30% ± 1% and 38% ± 1% (mean ± SEM), respectively. **(C_2_)** The number of filopodia per growth cone was unchanged by CNP application. Three independent experiments. 170 growth cones (control) and 177 growth cones (CNP) were assessed. *p* < 0.0001 (^∗∗∗^), *p* < 0.05 (^∗^), or *p* > 0.05 (ns), unpaired **(B_1_,C_1_,C_2_)** and paired **(B_2_)** two-tailed *t*-test. Error bars represent SEM. Scale bar, 20 μm.

FIGURE S2 Analysis of the specificity of rabbit anti-cGKI antibodies. Explants culture of embryonic DRGs on PDL/laminin1 were stained with rabbit anti-cGKI (0.75 μg/ml, IgG fraction) or anti-α-tubulin. **(A_1_–A_3_)** DRG explant from wild type cGKI mice. **(B_1_–B_3_)** DRG explant from cGKI-deficient mice. **(C_1_–C_3_)** Growth cone from wild type mice. **(D_1_–D_3_)** Growth cone from cGKI-deficient mice. No signals were detected for anti-cGKI in cGKI-deficient DRGs. Scale bar, 10 μm. Phall., phalloidin.

FIGURE S3 Localization of cGKI in the central domain of chick DRG growth cones cultivated on laminin1. **(A_1_)** α-tubulin, **(A_2_)** cGKI and **(A_3_)** α-tubulin and F-actin (with phalloidin), **(A_4_)** α-tubulin and cGKI. **(B_1_,B_2_)** Intensity profiles of cGKI and F-actin (phalloidin). Scale bar, 10 μm.

FIGURE S4 Colocalization of cGKI with marker proteins of intracellular compartments in DRG-derived F11 cells. **(A1–A4)** Colocalization between cGKI and the ER marker PDI in F11 cells. **(B_1_–B_4_)** Colocalization between cGKI and the lysosomal marker LAMP-1. **(C1–C4)** Colocalization between cGKI and the cis-Golgi-marker GM-130. Enlargement of the squares in **(A_3_–C_3_)** are shown in **(A_4_–C_4_)**. **(D)** Quantification of co-localizations using Mander’s colocalization coefficient (MCC). MCC values are given within the columns. tM1 value represents MCC for cGKI versus marker and tM2 represents marker versus cGKI. For GM130 18 cells, for TGN130 18 cells, for γ-adaptin 14 cells, for PDI 16 cells, for Rab11 18 cells and for LAMP-1 10 cells were analyzed. Error bars represent SEM. Scale bars, 20 μm.

FIGURE S5 Biorthogonal labeling strategy of palmitoylated proteins in growth cones of DRG neurons and in F11 cells. **(A)** DRG or F11 cells were grown overnight in standard cell culture medium followed by a medium without FCS supplemented with a palmitate analog containing an azide. The acyl synthase couples the palmitate analog to CoA followed by incorporation into proteins through zDHHCs palmitoyltransferases. Labeled proteins were detected by biotin-PEG4-alkyne and the click reaction. Adapted from (Gao and Hannoush, 2014). **(B)** Titration of F11cells incubated overnight with different concentrations of palmitic acid azide followed by the biorthogonal labeling procedure. 100 μM of palmitic acid azide was found to be appropriate as also suggested by others (Gao and Hannoush, 2014).

FIGURE S6 RT-PCR analysis of the mRNA expression of 3-hit candidates in E12.5 mouse DRGs. RT-PCR analysis of 3-hit candidates detected in the membrane fraction of F11 cells in E12.5 DRGs (DRG) and in E17 whole mouse embryo cDNA libraries (positive control, + ). Note that the third lane was loaded with water as control (Ctrl). All candidates appeared to be expressed at the mRNA level in mouse embryonic DRGs. Ctrl: water control. N/A, not attributed.

TABLE S1 Antibodies and dilutions.

TABLE S2 2-hit and 3-hit palmitoylated protein candidates which were detected only in the membrane samples of the F11 cells stimulated with a cGMP analogue are listed. 3-hit candidates are in bold.
