# Supplementary figures and images for: S-palmitoylation Is Required for the Control of Growth Cone Morphology of DRG Neurons by CNP-Induced cGMP Signaling
Source: Front Mol Neurosci. 2018 Sep 24;11:345. doi: 10.3389/fnmol.2018.00345 (PMC6166100; doi:10.3389/fnmol.2018.00345)

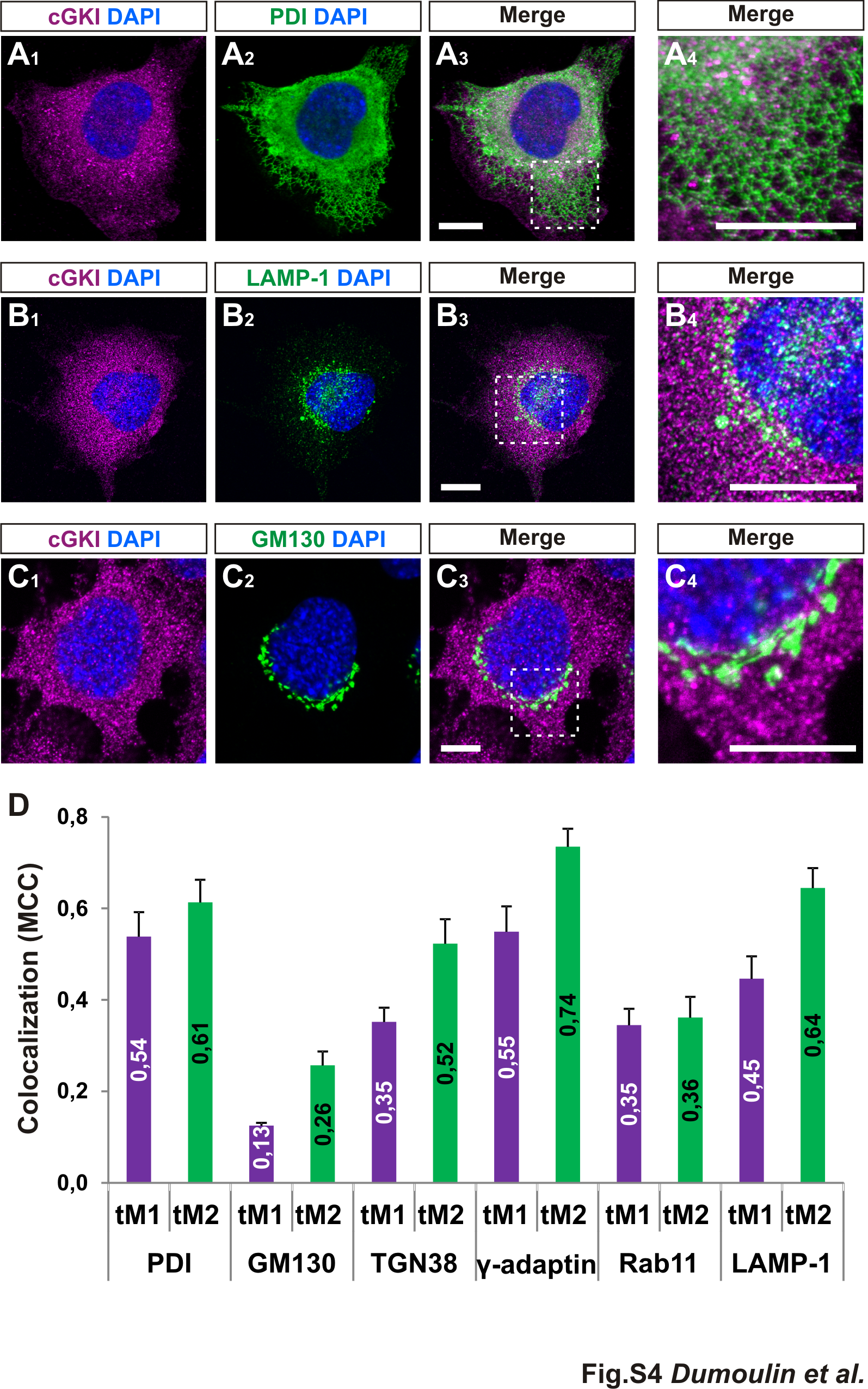

Supplement: FIGURE S4 — Colocalization of cGKI with marker proteins of intracellular compartments in DRG-derived F11 cells. (A1–A4) Colocalization between cGKI and the ER marker PDI in F11 cells. (B1–B4) Colocalization between cGKI and the lysosomal marker LAMP-1. (C1–C4) Colocalization between cGKI and the cis-Golgi-marker GM-130. Enlargement of the squares in (A3–C3) are shown in (A4–C4). (D) Quantification of co-localizations using Mander’s colocalization coefficient (MCC). MCC values are given within the columns. tM1 value represents MCC for cGKI versus marker and tM2 represents marker versus cGKI. For GM130 18 cells, for TGN130 18 cells, for γ-adaptin 14 cells, for PDI 16 cells, for Rab11 18 cells and for LAMP-1 10 cells were analyzed. Error bars represent SEM. Scale bars, 20 μm. [file Image_4.TIF]

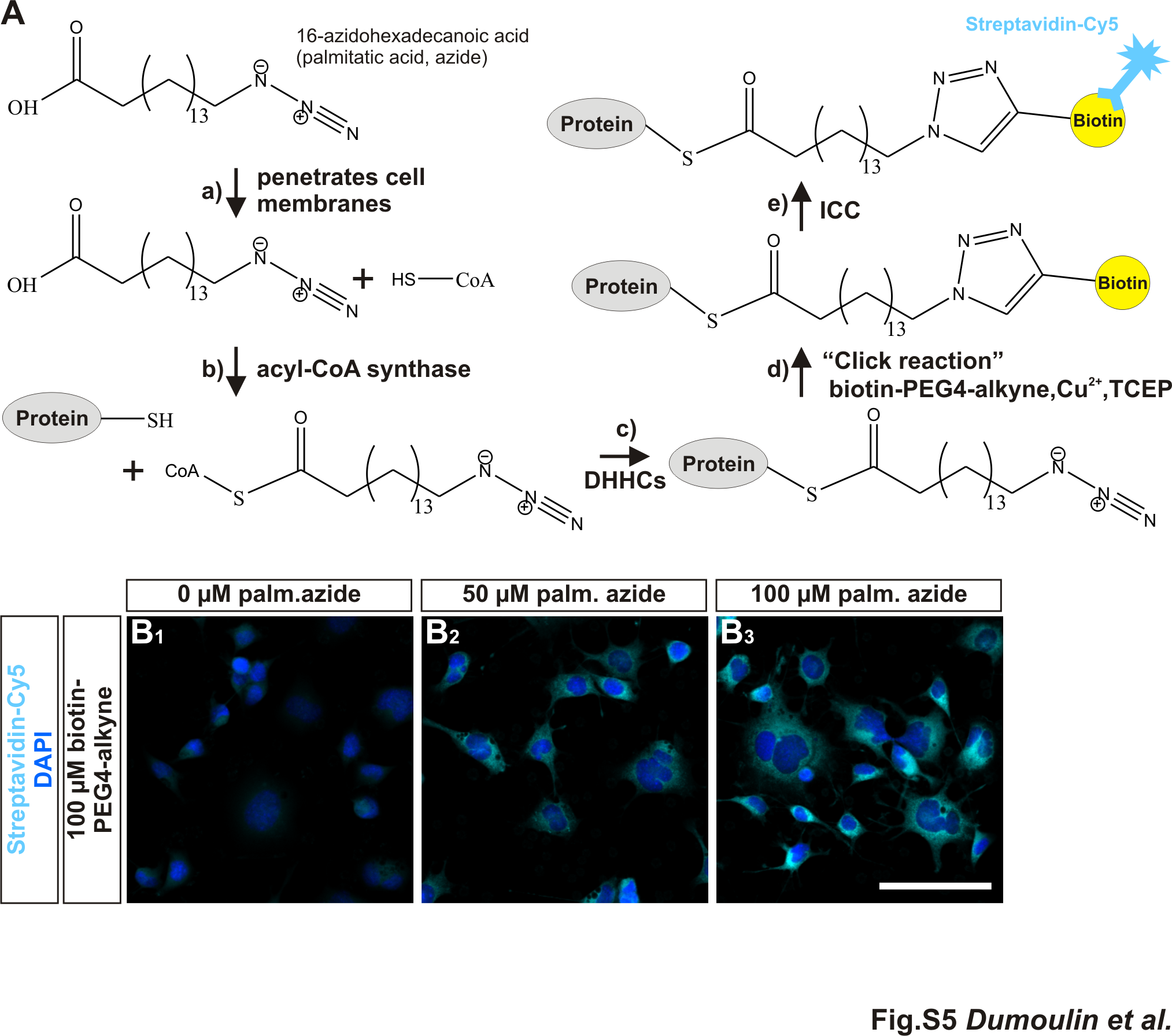

Supplement: FIGURE S5 — Biorthogonal labeling strategy of palmitoylated proteins in growth cones of DRG neurons and in F11 cells. (A) DRG or F11 cells were grown overnight in standard cell culture medium followed by a medium without FCS supplemented with a palmitate analog containing an azide. The acyl synthase couples the palmitate analog to CoA followed by incorporation into proteins through zDHHCs palmitoyltransferases. Labeled proteins were detected by biotin-PEG4-alkyne and the click reaction. Adapted from (Gao and Hannoush, 2014). (B) Titration of F11cells incubated overnight with different concentrations of palmitic acid azide followed by the biorthogonal labeling procedure. 100 μM of palmitic acid azide was found to be appropriate as also suggested by others (Gao and Hannoush, 2014). [file Image_5.TIF]
