# Supplementary material for: S-palmitoylation Is Required for the Control of Growth Cone Morphology of DRG Neurons by CNP-Induced cGMP Signaling
Source: Front Mol Neurosci. 2018 Sep 24;11:345. doi: 10.3389/fnmol.2018.00345 (PMC6166100; doi:10.3389/fnmol.2018.00345)
Supplement: TABLE S1 — Antibodies and dilutions. [file Table_1.docx]

**Table S1: Antibodies and dilutions.**

| **Name** | **Origin** | **Dilution** |
| --- | --- | --- |
| DAPI | Dianova | 1:1000 (IHC and ICC) |
| G-anti-M-488 | Jackson Immunoresearch (# 115-545-003) | 1:400 (IHC and ICC) |
| G-anti-M-647 | Dianova (#115-605-003) | 1:400 (ICC) |
| G-anti-M-HRP | Dianova (#GAM/IgG(H+L)/PO) | 1:25000 (WB) |
| G-anti-Rb-Cy3 | Jackson Immunoresearch (#111-545-003) | 1:400 (IHC and ICC) |
| G-anti-Rb-HRP | Dianova (#111-035-003) | 1:25000 (WB) |
| M-anti-GM130 (clone 35/GM130) | BD biosciences (#610822) | 1:250 (ICC) |
| M-anti-NeuN | Millipore (#MAB377) | 1:500 (ICC) |
| M-anti-NF-M (clone 2H3) | DSHB (#2H3) | 1:192 (IHC and ICC) |
| M-anti-PDI (clone 1D3) | Enzo (#ADI-SPA-891-D) | 1:500 (ICC) |
| M-anti-Rab11 | BD biosciences (#610656) | 1:100 (ICC) |
| M-anti-Rab5 (clone D11) | Santa Cruz (#sc-46692) | 1:500 (ICC) |
| M-anti-TGN38 | BD biosciences (#610899) | 1:800 (ICC) |
| M-anti-α-tubulin (clone B-5-1-2) | Sigma (#T5168) | 1:1000 (ICC) |
| M-anti-γ-adaptin | BD biosciences (#610386) | 1:250 (ICC) |
| Phalloidin-488 | Thermo Scientific (#A12379) | 1:100 (ICC) |
| Rb-anti-cGKI (clone 107) | (Ter-Avetisyan et al., 2014) | 1:1200 (IHC, ICC) |
| Rb-anti-chNCAM | (Rathjen and Rutishauser, 1984) | 1:4800 (ICC) |
| Rb-anti-Gap43 | Chemicon (#AB5220) | 1:1000 (WB) |
| Rb-anti-L1CAM | (Rathjen and Schachner, 1984) | 1:3000 (ICC) |
| Rb-anti-M-Cy3 | Jackson Immunoresearch (#315-545-003) | 1:400 (IHC and ICC) |
| Rt-anti-LAMP-1 (clone 1D4B) | Santa Cruz (#sc-19992) | 1:250 (ICC) |
| Streptavidin-Cy5 | Thermo Scientific (#SA1011) | 1:600 (ICC) |
