# Supplementary material for: S-palmitoylation Is Required for the Control of Growth Cone Morphology of DRG Neurons by CNP-Induced cGMP Signaling
Source: Front Mol Neurosci. 2018 Sep 24;11:345. doi: 10.3389/fnmol.2018.00345 (PMC6166100; doi:10.3389/fnmol.2018.00345)
Supplement: TABLE S2 — 2-hit and 3-hit palmitoylated protein candidates which were detected only in the membrane samples of the F11 cells stimulated with a cGMP analogue are listed. 3-hit candidates are in bold. [file Table_2.docx]

**Table S2:** 2-hit and 3-hit palmitoylated protein candidates which were detected only in the membrane samples of the F11 cells stimulated with a cGMP analogue are listed. 3-hit candidates are in bold.

| **Function** | **Gene Name** | **Function** | **Gene Name** |
| --- | --- | --- | --- |
| ***Channels and transporters:*** |  | ***Cell adhesion:*** |  |
|  | **Atp2c1** |  | Cadm4 |
|  | **Mcoln1** | ***Signalling, receptors:*** |  |
|  | **Slc25a13** |  | Clcn3;Clcn4;Clcn5 |
|  | Slc30a5 | ***Signalling, GTPases and other regulators:*** |  |
| ***Cytoskeletal proteins:*** |  |  | **Arhgef2** |
|  | **Map4** |  | Aldoa;Aldoart2;Aldoart1 |
| ***Metabolism:*** |  |  | **Atl2** |
|  | Abcb7 |  | Aatk |
|  | Alg3 |  | Pi4k2a |
|  | **Dad1** |  | Pde10a |
|  | Dolpp1 | ***Receptors and channels:*** |  |
|  | Edem3 |  | Cacng3 |
|  | Ephx1 | ***Others:*** |  |
|  | **Gfpt1** |  | Tm9sf2 |
|  | Glce |  | **Fv4** |
|  | Gusb |  | Tmem214 |
|  | Mogs |  | Efr3b |
|  | **Ndst1** | ***Unknown function:*** |  |
|  | Plod1 |  | Tmem222 |
|  | Prdx1 |  | Casd1 |
|  | Ptgs1 |  | Zfp493 |
|  | **Zdhhc13** |  | Ano8 |
|  | **Zdhhc3** |  | Bzw2 |
| ***Transcription and translation:*** |  |  | Zfp362;Zfp384;Znf384 |
|  | **Rps16** |  | C78339 |
|  | Eif2s1 | ***Known or putative false-positives:*** |  |
|  | Kdm1a |  | Acta1;Actc1;Actg2;Acta2 |
|  | Gcn1l1 |  | Sdhc |
|  | **Tars** |  | **Dpysl3** |
|  | Pes1 |  | **Dpysl2** |
|  | Nacad |  | **Uba1** |
|  | Nsun2 |  | **eno1** |
|  | Srbd1 |  |  |
|  | Eif2ak3 |  |  |
| ***Membrane trafficking:*** |  |  |  |
|  | Yipf5 |  |  |
|  | Trappc3 |  |  |
|  | Tmed10 |  |  |
